# Properties and Pharmacology of Scorpion Toxins and Their Biotechnological Potential in Agriculture and Medicine

**DOI:** 10.3390/toxins17100497

**Published:** 2025-10-07

**Authors:** Cháriston André Dal Belo, Stephen Hyslop, Célia Regina Carlini

**Affiliations:** 1 Departamento Multidisciplinar, Escola Paulista de Política, Economia e Negócios (EPPEN), Universidade Federal de São Paulo (UNIFESP), Rua General Newton Estilac Leal, 932, Quitaúna, Osasco 06190-170, SP, Brazil; 2 Departamento de Farmacologia, Faculdade de Ciências Médicas, Universidade Estadual de Campinas (UNICAMP), Rua Vital Brazil, 80, Cidade Universitária Zeferino Vaz, Campinas 13083-888, SP, Brazil; hyslop@unicamp.br; 3 Programa de Pós-Graduação em Biociências, Universidade Federal de Ciências da Saúde de Porto Alegre (UFCSPA), Rua Sarmento Leite 245, Porto Alegre 90050-170, RS, Brazil; 4 Centro de Biotecnologia e Departamento de Bioquímica, Universidade Federal do Rio Grande do Sul (UFRGS), Porto Alegre 91501-970, RS, Brazil

**Keywords:** insecticides, ion channels, neurotoxins, peptides, scorpion toxins, therapeutic applications

## Abstract

Scorpion venoms contain a wide range of toxins that interact with a variety of target molecules (ion channels, receptors and enzymes) associated with synaptic transmission, action potential propagation, cardiac function, hemostasis and other physiological systems. Scorpion toxins are also active towards bacteria, viruses, fungi and parasites. Such interactions make scorpion toxins useful lead molecules for developing compounds with biotechnological and therapeutic applications, and as tools for cell biology. In addition, scorpion toxins act as insectotoxins, with promising applications as insecticides. This review describes the range of scorpion toxins and discusses their usefulness for the development of insecticides and therapeutic drugs.

## 1. Introduction

**Figure 1:**
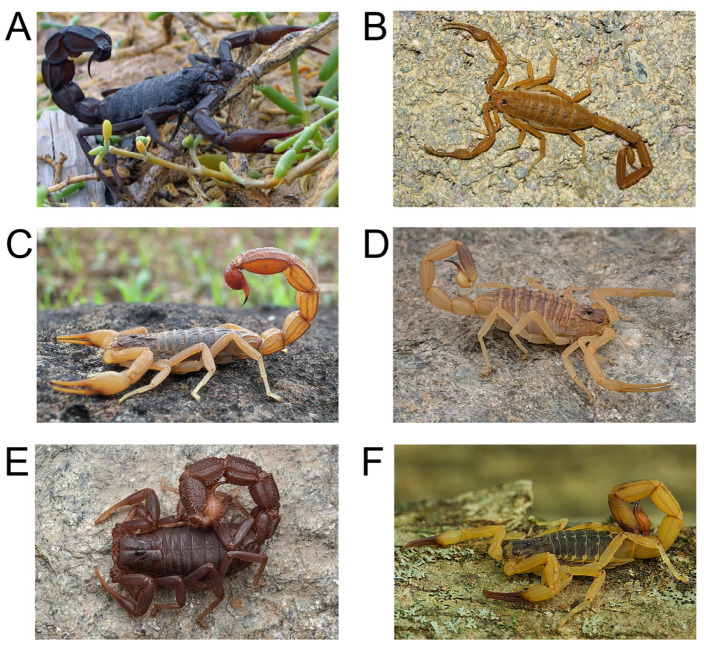
Representative scorpion species from around the world. (**A**). *Androctonus crassicauda* (Arabian fat-tailed scorpion; North Africa and Middle East). (**B**). *Centruroides sculpturatus* (Arizona bark scorpion; North America). (**C**). *Hottentotta tamulus* (Indian red scorpion; Indian subcontinent). (**D**). *Leiurus quinquestriatus* (Deathstalker scorpion; North Africa, Middle East and western Asia). (**E**). *Parabuthus transvaalicus* (South African thick-tailed scorpion; Southeastern Africa). (**F**). *Tityus serrulatus* (Brazilian yellow scorpion; South America). Photographs from Wikimedia Commons.

Scorpions (phylum Arthropoda, class Arachnida, order Scorpiones) are a highly diverse assemblage of approximately 2900 species classified into 24 families [1]. Scorpions have a worldwide distribution, but are absent from many temperate countries, certain Pacific islands and Antarctica [2]. The greatest diversity of scorpions occurs in tropical and subtropical regions and coincides with the distribution of most cases of scorpionism worldwide [3,4]. This extensive species diversity and wide geographic distribution attest to the biological adaptability and ecological success of scorpions [5]. Figure 1 shows examples of medically important scorpion species from around the world.

## 2. Scorpion Venoms: An Overview of Composition and Diversity

Scorpion venoms are a complex mixture of metal ions, amino acids and amines (e.g., serotonin), and low molecular mass peptides, neurotoxins, and enzymes such as hyaluronidase, metalloproteinases, and phospholipases A_2_ (PLA_2_) [6,7,8,9,10] that serve to capture and subdue prey and to provide defense against predation.

Numerous transcriptomic and proteomic studies over the last 20 years [11,12,13,14,15,16,17] have revealed the complexity of scorpion venoms, particularly with regard to their peptide content [10,18,19,20]. These peptides have been extensively studied because of their structural diversity, their wide-range of pharmacological effects, and their potential to serve as pharmacological tools, such as ion channel [21,22,23,24] and G-protein-coupled receptor [25,26] modulators, as well as inhibitors of enzymatic activity, including angiotensin-converting enzyme (ACE) [27,28,29,30,31,32,33,34] and matrix metalloproteinases [35]. Scorpion peptides have also been investigated as lead compounds for the development of novel therapeutic agents [10,36,37,38,39,40] to treat conditions such as bacterial [41,42,43,44,45] and viral [46,47] infections, cancer [47,48,49,50,51,52], pain [53,54], inflammation [39,55], diabetes [56] and hypertension [25,57,58]. In addition to peptides, scorpion venom enzymes, such as PLA_2_ [59,60] and hyaluronidases [61] have been studied for potential therapeutic applications.

## 3. Evolving Paradigms in Scorpion Toxin Classification

### 3.1. General Classification of Toxin Families

**Figure 2:**
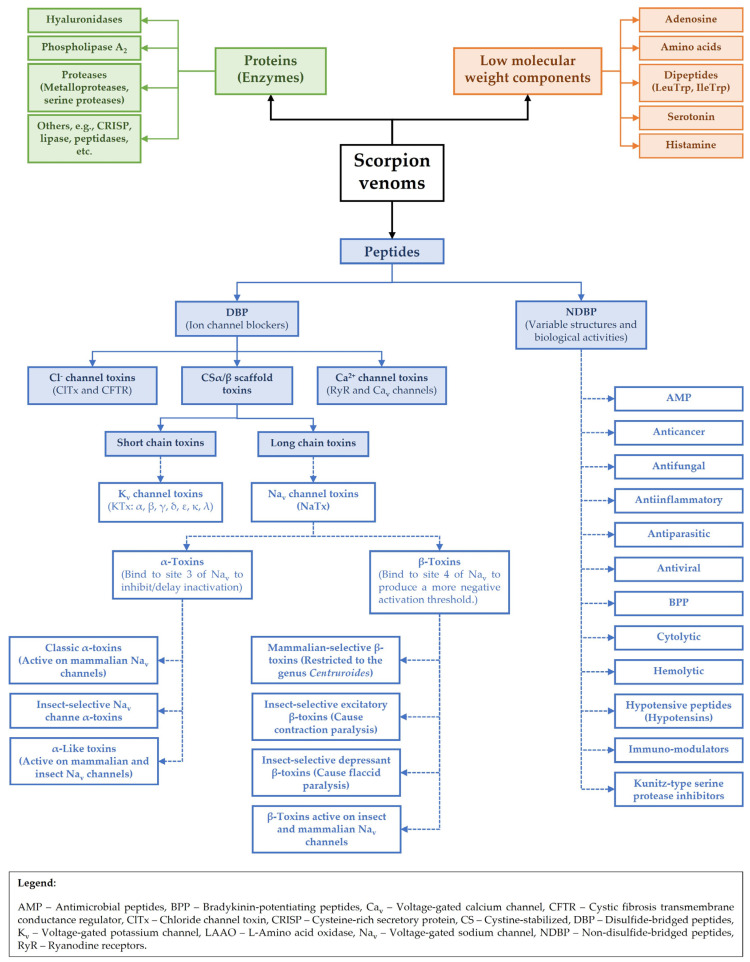
A general classification of scorpion venom components consisting of proteins (including several enzymes; green pathway), peptides with and without disulfide bonds (blue pathway), and low molecular mass compounds such as amines and amino acids (orange pathway). Of these components, DBP, consisting primarily of ion channel toxins, have been the most extensively studied, followed by NDBP and, in recent years, proteins (enzymes). The wide range of biological activities associated with NDBP reflects the marked structural diversity of these peptides. For specific details on the various groups of toxins, see the following Sections and corresponding tables (where applicable): Section 3.3 for DBP, Section 3.4 and Section 3.5 for NDBP, and Section 3.6 for proteins. For DBP subclasses, see Section 3.3.1 for CSα/β scaffold toxins (Na^+^ and K^+^ channel toxins are summarized in the Sections on Long-Chain Scorpion Toxins (Protein Family PF14866) and Short-Chain Scorpion Toxins (Protein Family PF00451), respectively), Section 3.3.2 for Ca^2+^ channel toxins and Section 3.3.3 for Cl^−^ channel toxins.

Historically, scorpion toxins have been classified based on their interactions with ion channels, particularly voltage-gated sodium (Na_v_), potassium (K_v_), calcium (Ca_v_), and chloride (ClC) channels, as a reflection of the central roles of these channels in neurophysiology and venom pathology, and also based on their primary amino acid sequences [7,19,20,21,22,23,24]. While this general approach is still applied today, the advent of high-throughput sequencing, structural biology techniques, and, more recently, artificial intelligence [62] has greatly expanded our understanding of toxin diversity such that scorpion toxins can now be classified based not only on their primary sequences, but also on their three-dimensional folding patterns and the conservation of key functional residues [54]. For instance, while many scorpion toxins have an α-helix/β-sheet fold configuration, variations in their disulfide bridge patterns and loop regions dictate their stability and their precise binding sites and pharmacological profiles [19]. This allows for the identification of new subfamilies within previously established groups and reveals a finer level of functional specialization, even among toxins targeting the same ion channel. This approach is particularly valuable for uncovering toxins with novel mechanisms of action or unusual target specificities. The primary general classification of scorpion peptide toxins includes (1) Disulfide-bridged peptides (DBP) and (2) Non-disulfide-bridged peptides (NDBP), although these venoms also contain peptides with other important biological functions [10]. Figure 2 provides a general classification of the principal components of scorpion venoms that includes proteins, peptides, and low molecular mass compounds.

### 3.2. General Organization of Scorpion Peptide Genes

Scorpion venom diversity is very high (estimated to be ~300,000 components across all species), with peptides being the principal components involved [63]. However, compared to the extensive biochemical and pharmacological characterization of numerous purified toxins, much less is known of scorpion toxins at the genomic level and of how variations in gene expression can influence venom composition and activity. While transcriptomic and proteomic studies have provided important insights into the general composition of scorpion venoms [11,12,13,14,15,16,17], detailed analyses of scorpion toxin gene organization and expression, and the possible types and mechanisms of post-translational modifications, have also been helpful in explaining the high diversity of toxins in these venoms at the genic level and the factors that influence this.

Scorpion peptide genes have a basic minimal organization consisting of coding regions for a signal peptide and the mature peptide of interest, often with the presence of introns in the signal peptide and mature peptide coding regions [64,65,66,67,68,69]. In some cases, a pro-peptide may also be coded [66,68,69], and such peptides, once removed, may persist in the venom, although it is unclear whether they exert any biological function [11,70]. Peptide toxins are generally coded for by their own specific genes rather than resulting from the post-translational processing of a large precursor protein to release mature peptides [64,65,66,68]. Specific events that can affect toxin diversity, in terms of sequence and biological activities, include single point mutations, deletions, insertions, alternative- and *trans*-splicing events, gene duplications, and post-translational modifications [17,67,71,72,73]. These mechanisms can lead to variations in venom peptide content and molecular size that can be detected by chromatographic analyses (especially RP-HPLC), mass spectrometry, immunoassays and other biochemical approaches, as well as biological assays.

Despite considerable advances in our knowledge of the structure and function of a wide variety of scorpion toxins in recent decades, for a much larger number of these toxins their phylogenetic and structural relationships with specific peptide or protein families, the occurrence of non-toxic homologs, their biological activities towards prey species, and their precise contribution to envenomation remain unclear. For the main toxin families discussed here, further details on their gene organization, protein sequences and structures, and biological activities are available through the UniProt Protein Family (PF) or Prosite (PS) codes indicated alongside the corresponding toxin groups, and from pertinent publications [12,64,65,66,68,69,74,75,76,77]. All peptide and protein sizes (indicated either as the number of amino acids or the molecular mass) mentioned in the main text and tables refer to the final size of the mature molecules, and not to precursor forms.

### 3.3. Scorpion Disulfide-Bridged Peptides (DBPs)

#### 3.3.1. Cystine-Stabilized α/β (CSα/β) Scaffold

This is the most prevalent and functionally diverse structural category of scorpion peptides and involves an α-helix connected to a β-sheet (often triple-stranded), stabilized by multiple disulfide bonds [78]. Most classic neurotoxins that target ion channels fall into this group. These molecules contain two completely conserved disulfide bonds at the Ci–Cj and Ci + 4–Cj + 2 positions, although some of them also exhibit an extra link connecting the two ends of the peptide chain [79]. These conserved disulfide bridges are vital for the structural integrity and biological activity of these peptides. By locking the structure into a precise and rigid three-dimensional conformation, these covalent bonds enable the peptide to effectively bind to its specific ion channel target and produce its characteristic pharmacological effects [80]. All scorpion peptides containing CS α/β motifs act in a similar way, namely, by interacting with ion channels to block or modulate normal channel function [36,81]. Members of this superfamily can be subdivided into long or short scorpion toxins, depending on their respective structures.

##### Long-Chain Scorpion Toxins (Protein Family PF14866)

These peptides are responsible for the severe neurotoxic effects observed in scorpion envenomation, primarily by disrupting the function of voltage-gated ion channels, particularly Na_v_ channels [36]. Their potency and specificity have made these toxins invaluable tools in neuropharmacology and drug discovery [82]. The typical gene organization for scorpion Na^+^ channel toxins (NaTx) includes two exons (exons 1 and 2) separated by an intron [64,65,66]. Exon 1 contains the 5′ non-coding region (untranscribed region, UTR) and the N-terminal region of the signal peptide, whereas exon 2 consists of the C-terminal of the signal peptide, the region coding the mature peptide, and the 3′ non-coding region. The overall premRNA size is 650–950 nucleotides with an intron of 300–620 nucleotides. Processing of this premRNA results in mature mRNA of 300–350 nucleotides that is translated to produce peptides of 82–96 amino acids from which the signal peptide (18–21 amino acids) is subsequently removed [64]. Further processing results in mature toxins of 60–76 amino acid residues in length [20,21,22,23,36,83,84]. The structural integrity and biological activity of NaTx are critically dependent on a conserved three-dimensional fold stabilized by three or, more commonly, four disulfide bridges [59]. The precise arrangement of these disulfide bonds and the configuration of specific loops within this fold dictate their specificity for different ion channel subtypes and their distinct mechanisms of action. Based on their electrophysiological effects on Na_v_, long-chain scorpion neurotoxins are broadly divided into two main functional classes, namely, α- and β-toxins. While both of these classes ultimately lead to neuronal hyperexcitability, they achieve this by binding to distinct receptor sites on the Na_v_ channel.

Alpha-toxins, often referred to as classic α-toxins, bind to receptor site 3 on the extracellular surface of Na_v_ channels, specifically interacting with elements in domains I and IV. Their primary effect is to inhibit or significantly slow the fast inactivation of these channels, thereby prolonging the open state of the channels, resulting in a persistent inward sodium current [85]. Physiologically, this action results in extended action potential duration and repetitive firing of neurons that causes hyperexcitability, muscle spasms, and paralysis [54]. AaH II from *Androctonus australis* Hector [86], BmK1 from *Buthus martensi* Karsch [87], CvIV4 from *Centruroides vittatus* [88], Lqh αIT from *Leiurus quinquestriatus hebraeus* [89] and Lqq III from *Leiurus quinquestriatus quinquestriatus* [90] are examples of scorpion α-toxins.

Beta-toxins bind to receptor site 4 of the Na_v_ channel, located within domain II. The primary effect of these toxins is to shift the voltage-dependence of activation to more negative (hyperpolarizing) potentials [91]. This means the Na_v_ channels become easier to open, with opening being possible even at resting membrane potentials, when the channels would normally be closed. This leads to spontaneous and repetitive action potentials and an increased excitability of nerve and muscle cells. Some β-toxins can also reduce the peak sodium current. Ts1 [92], Ts2 [93] and TsTX-I [94] from *Tityus serrulatus*, Cn2 [95] and Cn12 [96] from *Centruroides noxius*, BmK IT2 [97] from *Buthus martensii* Karsch, and Lqh-dprIT(3) [98] from *Leiurus quinquestriatus hebraeus* are representatives of scorpion β-toxins. However, not all NaTxs fit neatly into this two-category system, e.g., AaH IT4, from *Androctonus australis hector*, that exerts both α and β-NaTx effects [79].

##### Short-Chain Scorpion Toxins (Protein Family PF00451)

Short-chain scorpion toxins, also known as K^+^ channel toxins (KTx), represent a diverse group of scorpion venom peptides, generally 30–40 amino acids long and typically containing 3–4 disulfide bonds [20,21,36,99]. The overall gene organization for KTx is similar to that for NaTx in that it consists of a 5′-UTR, a signal peptide coding region, a mature peptide coding region and a 3′-UTR [12,64,65,68]. Introns are frequently observed in the C-terminal portion of the signal peptide coding region and can vary in size from relatively small, e.g., 78–94 bp for BmKTX, BmTX1, and BmTX2 [75] and BmP01, BmP03 and BmP05 [74], to quite large (500 bp) for BmKαTx11 and BmKαTx15 [100]. However, there is considerable variation in the location of introns among the different types of KTx. For example, the genes for α-KTx, κ-KTx, and γ-KTx, such as BmKTX (from *B. martensii*), BmKK7 (*B. martensii*), and HeTx203 (*Heterometrus spinifer*), respectively, all have a single intron that interrupts the signal peptide coding region [68]. For β-KTx, the signal peptide coding region may contain two introns, e.g., TtrKIK (*Tityus trivittatus*), or none; in the latter case, the intron (887 bp) is located in the region coding for the mature peptide, e.g., BmTXKβ2 (*B. martensii*), while for δ-KTx (toxins with a Kunitz-type fold) that also block KTx, such as LmKTT-1a (*Lychas mucronatus*) and BmKTT-2 (*B. martensii*), there are two introns in the mature peptide coding region and none in the signal peptide coding region [66,68]. In all cases, removal of the signal peptide (~20–28 amino acids) results in mature peptides ranging in size from 23 to 75 amino acids, depending on the subclass of KTx involved (Table 1) [12,68,74,75,76,100]. Some of these peptides may subsequently undergo post-translational modifications at the N-terminal, e.g., Gln modified to pyro-Glu in BmTX1 and BmTX2, and C-terminal amidation following removal of the terminal Gly in BmKTX [75].

KTxs exert a wide range of pharmacological activities through their ability to selectively block different K^+^ channel subtypes [101], including voltage-gated K^+^ channels (K_v_) [102] and Ca^2+^-activated K^+^ channels (K_Ca_) [103]. With the exception of the peptide MeuTXKβ1 from *Mesobuthus eupeus* venom [104], no other scorpion toxins capable of modulating two-pore-domain potassium (K2P) channels have been identified [105]. Several families and subfamilies of KTxs are recognized, based on their amino acid sequences, structure, and specific ion channel targets [23], with the main subfamilies being α, β, γ, κ, δ, λ, and ε-KTx [63,101,106,107,108].

Alpha-KTx, which generally contain 23–42 amino acid residues and three or four disulfide bridges, are potent blockers of K_v_ channels, with high selectivity for the subtypes K_v_1.1, K_v_1.2 and K_v_1.3 [102,109]. Beta-KTx consist of approximately 45–75 amino acid residues and two disulfide bridges [106,110] and also target various K_v_ channels, including K_v_1.3, K_v_4.2, K_v_4.3, and K_v_1.7 [81]. However, studying the modulation of K_v_ by these toxins is challenging because of their cytolytic effect [111]. Gamma-KTxs, with 35–45 amino acid residues and 3–4 disulfide bridges [106], occur in the genera *Centruroides*, *Mesobuthus*, and *Buthus*, and mainly block human Ether-à-go-go-Related Gene (hERG) channels [112,113,114]. Toxins with cysteine-stabilized helix-loop-helix folding (CSα/α) are classified as part of the κ-KTx family (25–30 residues and two disulfide bridges) [115,116]. Delta-KTx, also known as Kunitz toxins, have about 60–70 residues and 3–4 disulfide bridges with a double-stranded antiparallel β-sheet flanked by an α-helix in both the C-terminal and N-terminal segments [117]. These toxins modulate both protease and K^+^ channel activities [117,118,119]. Finally, the λ-KTx (35–40 residues, three disulfide bridges) and ε-KTx (29 residues, four disulfide bridges) families are characterized by a shared inhibitory cystine knot (ICK) motif [107]. The ε-KTx subfamily includes Ts11 and Ts12 isolated from *Tityus serrulatus* venom [107]. Although Ts11 shows less than 50% identity with KTxs from other subfamilies, it shares the ICK motif found in λ-KTxs, but is distinguished from λ-KTxs by possessing four disulfide bonds, whereas λ-KTxs have only three [23]. Table 1 summarizes the general properties of short-chain (KTx) toxins. Overall, the diversity of K^+^ channel subtypes and the specificity of the scorpion toxins acting on them indicates that these molecules have considerable therapeutic potential [21].

**Table 1:** Comparison of the main characteristics of the subfamilies of scorpion short-chain potassium channel-blocking toxins (KTxs).

KTx Subfamily	Length (Amino Acids)	Disulfide Bridges	Key Structural Features	Primary Target/Function	Noteworthy Characteristics	References
General Short KTxs	23–64	3–4	-	K^+^ channel blockade	-	[101]
α-KTx	23–42	3 or 4	CS α/β motif (α-helix + β-sheet)	K^+^ channel blockade	Largest subgroup of short scorpion toxins.	[63,120]
β-KTx	50–75	-	CS α/β motif	K^+^ channel blockade	Comprises longer chain peptides within the short toxin family.	[63,110]
γ-KTx	-	-	CS α/β motif	Human Ether-à-go-go-Related Gene (hERG) channels	Found in the genera *Centruroides*, *Mesobuthus*, and *Buthus*.	[112,121]
κ-KTx	-	2	Two parallel short α-helices connected by a β-turn	K^+^ channel blockade	Interaction with K^+^ channels similar to α-KTx, despite structural difference.	[115]
δ-KTx	59–70	3	Kunitz-type structural fold (double-stranded antiparallel β-sheet flanked by α-helix)	K^+^ channel blockade; Serine protease inhibition	Exerts antiprotease and K^+^ channel-blocking properties.	[68]
λ-KTx	29–49	3	Inhibitor cystine knot (ICK) motif (triple-stranded antiparallel β-sheet)	-	Related to calcins	[122,123]
ε-KTx	-	4	ICK motif (unique pattern)	-	Only two members (Ts11 and Ts12 from *T. serrulatus* venom); Ts11 shows <50% identity with other KTxs.	[23,107]

#### 3.3.2. Calcium Channel-Modulating Peptides (Calcins) (Protein Family PF08099)

Calcium channel-modulating peptides or calcins are relatively short peptides, typically containing 33–35 amino acids [124], that primarily affect ryanodine receptors (RyRs), intracellular calcium channels found in the endoplasmic/sarcoplasmic reticulum membrane [19,23]. Thus, whereas many scorpion toxins target K_v_ or K_Ca_ channels in the plasma membrane, calcins specifically target intracellular RyRs to modulate calcium release from internal stores rather than directly affecting the membrane potential or action potential firing as do KTx [23]. Well-known examples of calcins include imperacalcin (imperatoxin A) from *Pandinus imperator* (the Emperor scorpion) [125], maurocalcin from *Scorpio maurus palmatus* [126], hemicalcin from *Hemiscorpius lepturus* [127], hadrucalcin from *Hadrurus gertschi* [128,129], opicalcin from *Opistophthalmus carinatus* (African yellow leg scorpion) [124], urocalcin from *Urodacus yaschenkoi* [109,130] and vejocalcin from *Vaejovis mexicanus* [131].

Few calcins have been studied at the genic level. Zhijian et al. [132] reported that the gene for BmCa1, a ryanodine Ca^2+^ channel toxin, consists of three exons separated by two introns, with one of the introns (72 bp) located at the end of the signal peptide coding region and the other much larger intron (1076 bp) located within the mature peptide coding region; both introns begin with GT and end with AC, an arrangement typical of other scorpion introns. Removal of the 27-amino acid signal peptide from the precursor protein yields the mature peptide with 37 amino acids and three disulfide bonds. This gene organization is similar to that of opicalcins (opicalcine 1 and 2), from *Opistophthalmus carinatus* [133]. In this case, the gene consists of three exons each coding for a specific region (signal peptide, propeptide, and mature peptide) and two intros (487 and 544 bp, located at the N-terminal portion of the propeptide and mature peptide regions, respectively). A cDNA for opicalcin coded for a 22 amino acid signal peptide, an 11 amino acid propeptide rich in negatively charged amino acids, and a 33 amino acid mature peptide. The gene organization described above is shared by a variety of calcins and other scorpion toxins with an ICK motif (see below) [122]. In contrast to this gene organization (and to that for NaTx, KTx and ClTx-like peptides), there are no introns in the genomic DNA sequence of the depressant insect toxin BmK AS (and its homolog BmK AS-1), which acts on skeletal muscle ryanodine receptors [134]. In this case, the region coding for the 19 amino acid signal peptide sequence is followed immediately by that coding for the 66 amino acid mature peptide.

Structurally, calcins are characterized by an ICK motif, also known as a cystine stabilized α-helix-loop-loop-motif, that involves three disulfide bonds (S-S linkages, typically CysI-CysIV, CysII-CysV, and CysIII-CysVI) to form the ICK. The ICK confers a highly stable three-dimensional conformation that is resistant to proteolytic degradation [107] and is fundamental for calcin stability and biological activity [123,135].

Ryanodine receptors are ligand-activated channels involved in the rapid intracellular release of Ca^2+^ from endoplasmic reticulum and sarcoplasmic reticulum [124], with this release being fundamental for excitation-contraction (EC) coupling in muscle cells and excitation-secretion coupling in neurons [136,137]. Calcins bind to an extraluminal (cytoplasmic) domain of the RyR [138] to allosterically induce conformational changes that facilitate or prolong the open state of the channel [139]. The high affinity and selectivity of calcines for specific RyR isoforms, e.g., RyR1, are key aspects of their pharmacological profile [138].

#### 3.3.3. Chloride Channel Toxins (Protein Subfamily PS51200)

**Figure 3:**
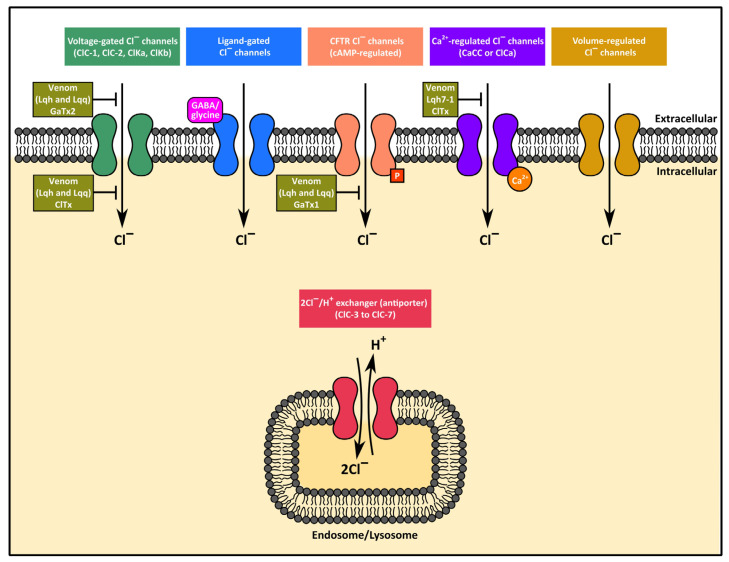
Diversity of chloride (Cl^−^) channels and the sites of action (extracellular, intracellular) of scorpion venoms and toxins that interact with these channels. See Table 2 for details of these interactions and toxin channel specificities. Lqh and Lqq venoms and GaTX1 have no effect on CFTR when applied to the extracellular surface. Note that the original studies by Debin and colleagues [143,144] only showed that ClTx blocked ClC from the cytoplasmic side, without identifying the specific channel type. Pure ClTx was subsequently shown not to block ClC-2 channels [145]. Note also that although ClTx has been shown to block Ca^2+^-regulated Cl^−^ channels [146], another study reported no such blockade [147]. CFTR—cystic fibrosis transmembrane conductance regulator, ClTx—chlorotoxin, GABA—γ-aminobutyric acid, Lqh—*L. q. hebraeus*, Lqq—*L. q. quinquestriatus*, P—phosphorylation.

Cell membrane permeability to chloride (Cl^−^) ions is mediated by a range of membrane proteins that include voltage-gated chloride channels (ClC-1, ClC-2, ClKa and ClKb), chloride/proton (2Cl^−^/H^+^)-exchangers (ClC-3 to ClC-7), chloride channels activated by an elevation in intracellular Ca^2+^ (CaCC or Cl_Ca_), volume-regulated chloride channels, ligand (γ-aminobutyric acid—GABA, glycine)-gated chloride channels, and the cystic fibrosis transmembrane conductance regulator (CFTR) that is modulated via phosphorylation by protein kinase A (cAMP-dependent protein kinase or PKA) and ATP hydrolysis [140,141,142] (Figure 3 [143,144,145,146,147]). Whereas most of these proteins function as channels located in the plasma membrane, the 2Cl^−^/H^+^-exchangers are antiporters (importing 2 Cl^−^ for every H^+^ exported) located primarily intracellularly, in endosomes and lysosomes [142]. Chloride channels conduct chloride ions (Cl^−^) and other anions (Br^−^, I^−^, HCO_3_^−^, NO_3_^−^, SCN^−^ and small organic acids) across the cell membranes of tissues such as cardiac and skeletal muscle, neurons, and intestinal, pulmonary and renal epithelia, and are involved in activities such as membrane repolarization (hyperpolarization) and stabilization, intracellular pH and volume regulation, and the regulation of electrolyte balance and fluid transport [140]. Mutations in these different channels can result in a variety of diseases, including congenital myotonias, cystic fibrosis (involving CTRF), deafness, epilepsy, osteoporosis, renal dysfunction (formation of kidney stones, salt-wasting and reduction in the volume of extracellular fluid), and other conditions [141,142,148].

Transcriptomic, proteomic and genomic analyses of scorpion venoms have indicated that toxins active on Cl^−^ channels are much less abundant than those acting on K^+^ and Na^+^ channels, generally accounting for <5% of venom peptides [17,149,150,151,152]. Structurally, Cl^−^ channel toxins are short-chain toxins (mature peptides have 35–37 amino acids) containing eight cysteine residues arranged in the consensus sequence …CXXC…CXXCC…C…CXC… that form four disulfide bonds [153], but few of these toxins, and from only two venoms (*L. q. hebraeus* and *L. q. quinquestriatus*), have been characterized electrophysiologically and pharmacologically in any detail (Table 2 [143,144,145,146,147,154,155,156,157,158]).

The few reports that have examined the gene structure of ClTx-like toxins indicate that the overall organization is similar to that for other scorpion peptide toxins, i.e., coding regions for the signal and mature peptides, with an intron (88–93 bp) located in the region coding for 24–25 amino acid signal peptides [65,77,159,160,161]. Thus, for example, the gene (364 bp) for Bm-12, a 35 amino acid ClTx-like peptide from *B. martensii* venom (identical to BmKCT cloned by Zeng et al. [160] and subsequently renamed as BmKClTx3 [77]), contains a 93 bp intron in the 24 amino acid signal peptide coding region; this intron size is similar to that of other short-chain toxins (80–100 bp), but smaller than for long-chain peptides such mammalian α-neurotoxin, neurotoxins that affect mammal and insects, and insect excitatory neurotoxins (introns > 400 bp) [159]. Mature ClTx-like peptides have no terminal glycine, thus precluding post-translational amidation [66,161].

Although several short-chain toxins, later found to be structurally related to ClTx, were identified in the 1970s and 1980s [153], DeBin and Strichartz [143] were the first to pharmacologically demonstrate the ability of a scorpion venom (*L. quinquestriatus*) to block slow conductance ClC of embryonic rat brain growth cones and rat colonic epithelial cells, with a stoichiometry of one toxin molecule per channel; the molecular mass of the toxin was estimated to be ~5 kDa. The toxin responsible for this blockade (chlorotoxin, ClTx) was subsequently purified and sequenced (molecular mass: 4.07 kDa, with eight cysteines that formed four disulfide bonds) and shown to account for ~4.3% of the venom protein content. ClTx selectively blocked open ClC in a voltage-dependent manner and produced progressive, reversible paralysis in crayfish (crustaceans) and cockroaches [146]. Nuclear magnetic resonance (NMR) experiments later defined the α-helical and β-sheet structure and disulfide bonds of ClTx [162]. Subsequent studies indicated that *L. quinquestriatus* venom contained a toxin(s) capable of blocking ClC-2 (but not CIC-0 or CIC-1) channels [145] and CFTR [155,156]. Since the initial description of ClTx, various peptides structurally related to ClTx have been described from other scorpion species [153], including AaCt (*Androctonus australis*) [163], BmKCT (*Buthus martensii* Karsch) [160], Bs-Tx7 (*Buthus sindicus*) [164], BtITx3 [165] and ButaIT [166] (both from *Buthus tamulus*), GaTX1 [157] and GaTX2 [154] (both from *L. q. hebraeus*), Lqh2-2, Lqh7-1 and Lqh8-6 (*L. q. hebraeus*) [158], meuCl14, meuCl15 and meuCl16 (*Mesobuthus eupeus*) [167], OdClTx1 (*Ondobuthus doriae*) [168], and Ce-1 (*Compsobuthus egyptiensis*) [169]. However, the pharmacology of most of these peptides remains unknown or poorly characterized and the extent to which they interact with Cl^−^ channels remains to be determined. ClTx itself is not a general Cl^−^ channel blocker as native toxin applied extracellularly does not block volume-regulated, Ca^2+^-regulated, CFTR (cAMP-regulated) Cl^−^ channels, and glioma-specific Cl^−^ channels [147,155]. GaTX1 selectively blocks CFTR [145,157] and Lqh7-1 blocks Ca^2+^-activated Cl^−^ channels in vascular myocytes [158]. An important limitation in the electrophysiological study of toxins such as ClTx and GaTX1 is that they are apparently active only when applied to the intracellular (cytosolic) surface of their target channels such that their true extracellular sites of action on ion channels remain to be determined [144,147,157].

**Table 2:** Scorpion venoms and toxins active on chloride (Cl^−^) channels.

Species	Toxin Name	Molecular Mass (kDa)	Target(s)	Biological Activity	References
*Leiurus quinquestriatus quintestriatus*	Venom (peptide(s) not identified)	-	Slow conductance Cl^−^ channels (ClC)	Venom caused voltage-dependent reversible blockade of small conductance Cl^−^ channels from embryonic rat brain growth cones (200 μg/mL) and anion channels from rat colonic epithelial cells (enterocytes) (100 and 400 μg/mL) reconstituted in artificial membranes (stoichiometry of one toxin molecule per channel). Channels blocked in open state. Large conductance Cl^−^ channels of blood cells (predominantly platelets and white blood cells) were unaffected by venom (200 μg/mL). Specific Cl^−^ channel type not identified in these studies.	[143,144]
	Chlorotoxin (ClTx)	~5 (4.07)	Slow conductance Cl^−^ channels (ClC)	Purified toxin (ClTx) reproduced the venom activity initially reported by DeBin and Strichartz [143] and blocked CIC with a K_D_ of ~600 nM. ClTx caused progressive, reversible paralysis (recovery depended on venom dose) in crayfish and cockroaches, and accounted for ~4.3% of the venom protein content. Blockade seen only when ClTx was applied to the cytoplasmic surface. A later study reported that ClTx applied extracellularly did not block volume-regulated, CFTR (cAMP-regulated) and glioma-specific Cl^−^ channels [147].	[144,147]
			Ca^2+^-regulated Cl^−^ channels	Ca^2+^-regulated Cl^−^ channels in astrocytes were potently blocked by ClTx [146], whereas similar channels in a human colon carcinoma (T84) cell line were not blocked by ClTx [147].	[146,147]
*Leiurus quinquestriatus hebraeus*	Venom (peptide(s) not identified)	----	ClC-2 Cl^−^ channels	A peptide-enriched fraction of venom (obtained by filtering through 10 kDa-cutoff filters) caused concentration-dependent, progressive, reversible blockade of rabbit CIC-2 (but not *Torpedo* CIC-0 or human CIC-1) channels when applied extracellularly. Inhibition was unaffected by boiling the venom fraction, but was prevented by incubation with trypsin, indicating involvement of a heat-stable peptide. Blockade produced by modulating the channel gating mechanism (slower activation, but unaltered deactivation). Purified ClTx from *L. q. quinquestriatus* was inactive on CIC-2 channels.	[145]
	GaTX2 (Gating modifier of anion channels 2)	3.2	ClC-2 Cl^−^ channels	A continuation of the investigation reported in [145] identified a toxin with three disulfide bonds and a sequence unrelated to ClTx but identical to previously identified leiuropeptide II from this same venom. Structurally related to toxins that inhibit K^+^ channels (apamin-sensitive K^+^ channels, Ca^2+^-activated K^+^ channels and K_v_1.2 channels). Causes voltage-dependent blockade of ClC-2 channels with an apparent K_D_ of ~20 pM. Slows channel activation but does not block open channels. No blockade of CIC-1, CIC-3 and CIC-4 channels or transporters, nor of CFTR (when applied extracellularly or intracellularly). No effect on GABA_C_ receptors when applied to the extracellular surface, or when applied to the cytosolic side of endogenous Ca^2+^-activated chloride Cl^−^ channels. No effect on K_v_1.2 channels.	[154]
	Venom (peptide(s) not identified)	----	Cystic fibrosis transmembrane conductance regulator (CFTR)	Initial experiments showed that venom reversibly inhibited the CFTR channel in a voltage-dependent manner via a pore-block mechanism. Rapid, all-or-none blockade involving high affinity interaction with the nucleotide binding site of the channel in an interburst closed state, with a reduction in channel burst duration and open probability. No effect on ATP-dependent macroscopic opening rate. Only active when applied to the cytoplasmic side of phosphorylated channels. Activity was unaffected by boiling but was abolished by incubating venom with trypsin, suggesting peptide involvement. No effect on *Xenopus* oocyte Ca^2+^-activated Cl^−^ channels (Cl_Ca_) when added to the extracellular or cytosolic side. Purfied ClTx from *L. q. quinquestriatus* was inactive on CFTR.	[155,156]
	Georgia anion toxin 1 (GaTX1)	3.67	CFTR	Potent, state-dependent (closed channel), reversible blockade of CFTR from the cytosolic side, with K_D_ = 41.5 nM and IC_50_ = 48 nM. Reduced the open probability and increased the closed time of the channel. Blockade was reduced by high [ATP]. Possibly acts as a non-competitive blocker of CFTR. Greater than 94% identity with cDNA-derived sequences of ClTx-a, -b and -c and >62% sequence identity with various other ClTx-like peptides. No effect when applied to the extracellular surface of CIC-1 and CIC-2 channels or the chloride/H^+^ exchanger (antiporter) CIC-3, or the cytoplasmic surface of CIC-2 channels. GaTX1 was therefore different from the venom peptide that inhibits CIC-2 channels (see above). At concentrations that affected CFTR, GaTX1 had no effect on GABA_C_ receptors when applied to the extracellular surface, or when applied to the cytosolic side of endogenous Ca^2+^-activated chloride Cl^−^ channels. Also did not affect the ABC transporters MRP1, MRP2, and MRP3.	[157]
	Lqh7-1	3.65	Ca^2+^-regulated Cl^−^ channels	Of three ClTx-related peptides identified (Lqh2-2, Lqh7-1 and Lqh8-6), Lqh7-1 blocked Ca^2+^-regulated Cl^−^ channels in rat portal vein myocytes (IC_50_ 63 ± 13 nM). Synthetic Lqh-1 caused similar blockade (IC_50_ 49 ± 5 nM). Lqh2-2 caused only 50% blockade at 1 μM, whereas Lqh8-6 was inactive. Lqh7-1 had no effect on L-type and T-type voltage-dependent Ca^2+^ channels, on intracellular Ca^2+^ release via ryanodine-sensitive channels, or on Ca^2+^-activated and voltage-activated K^+^ currents.	[158]

Only toxins for which electrophysiological studies on Cl^−^ channels have been reported are listed here. As indicated in the main text, various ClTx-related peptides have been identified in scorpion venoms, but very few of these have been rigorously examined with regard to their ion channel pharmacology. ABC—ATP-binding cassette, MRP—multidrug resistance-associated protein.

The biological functions of Cl^−^ channel toxins present in scorpion venoms remain largely unknown, mainly because few studies have examined the action of these peptides on the natural prey of most scorpion species. A few studies have examined the effects of these toxins on invertebrates. ClTx is paralytic in crayfish (*Procambarus clarkii*) and cockroaches (*Periplaneta americana*) [144], and lethal to aphids (*Acyrthosiphon pisum*) [153], while structurally related peptides (I1, I3, I4, and I5) from *M. eupeus* cause paralysis in *Nauphoeta cinerea* cockroaches [153] and peptides BTChl2 [144] and BtITx3 [165] from *B. tamulus* are lethal to tobacco bollworms (*Heliothis virescens*) and cotton bollworms (*Helicoverpa armigera*), respectively. Peptide ButaIT, also from *B. tamulus*, causes selective progressive irreversible flaccid paralysis of *H. virescens* but is not toxic to blowfly larvae or mice [166], while Ce-1 from *Compsobuthus egyptiensis* is lethal to crickets (*Acheta domesticus*) [169]. Although Cl^−^ channel modulation is assumed to be involved in the physiological effects of these toxins in invertebrates, especially insects, the precise molecular mechanisms involved remain unknown.

ClTx remains the best-characterized Cl^−^ channel toxin from scorpion venoms [170] and, over the years, has been proposed as a potentially useful molecule for treating certain tumors [171,172], particularly brain gliomas [173,174,175,176]. More recently, conjugates containing ClTx have been developed for imaging, diagnosing and treating glioblastoma brain tumors [51,177,178,179]. Interactions of ClTx with ClC-3 channel proteins and a variety of non-channel proteins, such as annexin 2, matrix metalloproteinase-2 (MMP-2), neuropilin 1 and others [164,180] may be important in the antitumor activity of ClTx and related peptides, possibly mediated via the inhibition of MMP-2 activity [153,181]. Indeed, such interactions, rather than Cl^−^ channel blockade, may be the most relevant physiological actions of ClTx [153,170].

### 3.4. Non-Disulfide-Bridged Peptides (NDBPs)

In addition to the abundant disulfide-bridged peptides described above, scorpion venoms also contain peptides (13–56 amino acids) lacking these bonds (non-disulfide-bridged peptides, NDBPs) [36,62,182,183,184,185]. The structural diversity of NDBPs has made their classification difficult, with the simplest classification being based on their linear amino acid sequence, i.e., short (<20 amino acids), medium (20–35 amino acids) and large (>35 acids) peptides [184], or a variation in this [45], with most NDBPs being short-chain peptides. Secondary structure analyses of these peptides indicate that, with few exceptions, e.g., Peptide T from *Tityus serrulatus* [27] and peptide K12 from *Buthus occitanus* [28] (both of which are bradykinin-potentiating peptides), NDBPs exist in a random coil conformation in aqueous solution, but form cationic, amphipathic α-helical structures under appropriate conditions, e.g., in dodecylphosphocholine (DPC) micelles [62,183]. Based on this core structure, NDBPs have been classified into three major groups: (1) Peptides with a completely α-helical structure, (2) Peptides with a central α-helical domain and random coils in the N and C terminal regions, and (3) Peptides with two α-helical regions separated by a central random coil region [156]. More comprehensive classifications include a combination of peptide length, sequence similarity and physiological functions [182,183].

The structural diversity and flexibility of NDBPs allows them to interact with a range of molecular targets and helps explain the diversity of biological activities among these peptides (Figure 2), with many of them having more than one activity [36,182,183,184]. However, few specific targets have been identified so far for NDBPs, with many of the biological actions reported for these peptides being rather general or non-specific. The finding that many of the biological activities of NDBPs, e.g., antibacterial, antiviral, antiparasitic and anticancer activities, involve interaction with negatively charged biological membranes probably reflects the cationic nature of most NDBPs (with charges ranging from +1 to +7, and isoelectric points > 9.5) [36,44,45,183].

### 3.5. Non-Channel-Modulating Peptides

In addition to the complex diversity of peptides that modulate ion channels, scorpion venoms also contain peptides with other biological activities not directly related to ion channel function. These include bradykinin potentiating peptides (BPPs) that inhibit ACE activity [27,28,29,30,31,32,33], peptides that potentiate the hypotensive activity of bradykinin by stimulating nitric oxide release rather than by ACE inhibition [25,57,58], peptides that modulate G-protein-coupled receptors [25,26], angiogenesis and inflammation [186], intracellular signaling pathways [187], and inhibit metalloproteinases [35], and a wide variety of peptides with antibacterial, antiviral, antiparasitic and other activities. This diversity of peptides and their range of activities can be increased by recombination events at the level of transcription [29,30,188] and by post-translational modifications [18,31,72,73]. As a result, a given peptide may exert a range of biological effects [29], further amplifying the molecular targets in vivo.

### 3.6. Enzymes

Most scorpion toxins studied to date have been peptides, but scorpion venoms also contain enzymes, particularly hyaluronidases, phospholipase A_2_ (PLA_2_), and proteases, particularly metalloproteases [189,190,191,192,193], although not all scorpion venoms contain these enzymes [190,194]. Hyaluronidases (spreading factor) are widely distributed among scorpion venoms [61,195,196,197,198,199] and facilitate the dispersion of toxins from the sting site through their ability to damage the surrounding extracellular matrix and connective tissue [200,201,202,203,204]. Hyaluronidase activity can be inhibited by antivenoms [195] and flavonoids [201]. These enzymes have various applications, such as in enhancing drug dispersion and in cancer therapy [203], and scorpion hyaluronidases could have similar uses, although this has not been extensively studied.

Venom PLA_2_, particularly those of snake venoms, are well-known for their ability to cause tissue necrosis, hemorrhage, hemolysis, coagulation disturbances, inflammation and pain [205,206,207]. These enzymes have been identified and characterized from several scorpion venoms [59,60]. In contrast to snake venom PLA_2_ that belong to groups I (Elapidae and Hydrophidae) and II (Viperidae) in the classification of PLA_2_, scorpion PLA_2_ belong to group III that includes PLA_2_ from bee, bumblebee, jellyfish and Mexican beaded lizard (*Heloderma horridum horridum*) venoms. Structurally, scorpion venom PLA_2_ have a conserved calcium-binding loop and active site when compared to snake venom PLA_2_, although the calcium-binding loop is closer to the N-terminal than in group I and II PLA_2_. Scorpion PLA_2_ differ from groups I and II and certain group III PLA_2_ in that they are heterodimeric enzymes with a long enzymatic chain containing the calcium-binding loop and active site that is linked by a disulfide bridge to a short chain. This dimeric structure is produced by the release of a pentapeptide from the proenzyme during maturation [59]. Although scorpion venom PLA_2_ exert a variety of biological activities in experimental studies in vitro and in vivo, including anti-angiogenic, anti-tumoral, anticoagulant, hemolytic, and inflammatory actions, and possibly cardiotoxicity and neurotoxicity [59,60], their precise role in scorpion envenoming remains to be established. Specifically, scorpion PLA_2_ may contribute to venom-induced inflammation and pain, as demonstrated for snake venom PLA_2_ [208,209,210,211]. As such, these enzymes could be therapeutically interesting targets for inhibition by small molecules such as varespladib [212].

Proteases (metalloproteases and serine proteases) have been identified in a variety of scorpion venoms [114,189,191,192,198,213,214,215,216,217,218,219,220,221]. Most metalloproteases have been identified based solely on transcriptomic and proteomic analyses, and very few have been purified and characterized biochemically and evaluated for their biological activities, e.g., antarease [222,223]. Metalloproteases may be involved in the maturation of toxin precursors through post-translational processing [224] and in the metabolism of biologically active peptides [225]. These enzymes may also contribute to venom diffusion from the sting site through their ability to degrade extracellular matrix proteins, in addition to exerting other effects such as inhibiting platelet aggregation, altering cytokine release, activating the complement cascade [226,227,228], as well as mediating inflammation and pain [229,230]. The extent to which metalloproteases contribute to and modulate the effects of scorpion venoms has not been extensively investigated. In this regard, the use of selective low-molecular mass metalloprotease inhibitors and drug repurposing could be therapeutically relevant in attenuating the actions of scorpion metalloproteinases in the local (edema, inflammation and pain) and systemic (coagulation disturbances, hemodynamic alterations and pulmonary edema) effects of envenoming, in a manner analogous to the situation with snake venoms [231,232,233,234,235,236,237,238].

## 4. Insecticidal Activity of Scorpion Venoms and Toxins

### 4.1. Insecticidal Potential

Globally, insect pests reduce agricultural yields by 10–16% before harvest and consume a similar proportion following harvest [239], with the largest food-producing countries (China and the United States) experiencing the highest losses from invasive insects [240]. Overall, agricultural losses to invasive insects cost at least US$70 billion per year globally, with associated health costs exceeding US$6.9 billion per year [241,242].

The use of natural and synthetic chemical insecticides has revolutionized the management of insects that affect human health, agriculture and natural resources. However, the continued and often indiscriminate use of these chemicals, besides triggering the development of resistance in different populations of target and non-target insect species [243], also leads to significant sublethal effects on these organisms. Such effects can impact their physiology and behavior, even at doses lower than those typically considered lethal [244,245,246]. The ongoing challenge of insect resistance to insecticides is further complicated by the discovery of non-traditional resistance mechanisms and highlights the need for advanced monitoring and management strategies [247,248,249].

Scorpion venoms have gained increasing attention for their potent insecticidal properties. The insecticidal activity of scorpion venoms primarily stems from their neurotoxic components that exhibit marked specificity towards insect neuronal channels, particularly voltage-gated sodium, potassium, and calcium channels. By disrupting the normal ion flow, these toxins induce paralysis and ultimately lead to insect death. For example, specific scorpion toxins can bind to and modify the gating kinetics of insect sodium channels, causing persistent activation and neuronal hyperexcitability [250]. In addition to neurotoxicity, some scorpion venom components are cytotoxic through their ability to disrupt the cell membrane, leading to cell lysis and death [251]. These toxins may also interfere with other cellular processes, such as mitochondrial function and signal transduction pathways [252].

**Figure 4:**
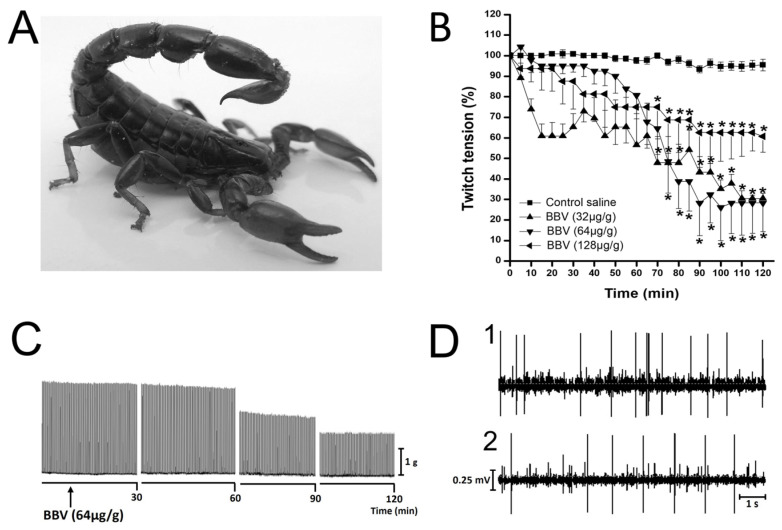
The neuromuscular and neuronal activity of *Bothriurus bonariensis* scorpion venom (BBV) in *Nauphoeta cinerea* cockroaches. (**A**) *Bothriurus bonariensis*. (**B**) Neuromuscular paralysis (seen as a decrease in muscle twitch-tension) caused by BBV in cockroach neuromuscular preparations. (**C**) Representative recording of cockroach neuromuscular twitches showing the blockade by BBV. (**D**) Extracellular recordings of spontaneous neural compound action potentials (SNCAP) from cockroach sensilla showing a decrease in the frequency of potentials after incubation with BBV (recording 2) compared with the saline control (recording 1). The points in (**B**) represent the mean ± SEM (n = 6). * *p* < 0.05 compared to saline controls. Modified from dos Santos et al. [253].

Insects are a primary food source for most scorpions and scorpion venoms contain insectotoxins with varying degrees of specificity towards different insect orders [253,254]. Examples of these insectotoxins include AaIT (*Androctonus australis*) [254], BjIT 1 and BjIT 2 (*Buthotus judaicus*) [255,256], and SmIT 1 and SmIT 2 (*Scorpio maurus palmatus*) [257]. Some toxins show broad-spectrum activity and affect a wide range of insects, while others display narrow selectivity towards specific pests. This specificity is crucial for developing targeted pest control strategies that minimize harm to non-target organisms. The potential applications of scorpion toxins in agriculture and public health are significant. For example, they could be used to control agricultural pests that damage crops, thereby reducing the need for synthetic pesticides [258]. Additionally, they could be used to combat disease-carrying insects, such as mosquitoes and flies, thereby contributing to public health initiatives [259]. Scorpion toxins have also been shown to be active against economically important pests such as lepidopteran larvae and dipteran vectors [64]. However, further research is needed to fully understand the mechanisms of action involved, identify specific toxins with high insecticidal activity, and develop safe and effective delivery systems. Furthermore, careful consideration of potential environmental impacts and the development of sustainable production methods are essential for achieving the full potential of scorpion toxins in pest control. Scorpion insectotoxins also have promising applications as insecticides. For example, the venom of the scorpion *Bothriurus bonariensis* (C.L. Koch, 1842) from southern Brazil (Figure 4A) exerts insecticidal activity in the lobster cockroach *Nauphoeta cinerea* [253]. In these cockroaches, the venom causes neuromuscular paralysis (Figure 4B,C) and has a depressant activity upon spontaneous neural compound action potentials (SNCAP) (Figure 4D). These pharmacological activities suggest the presence of insectotoxins capable of interacting with voltage-gated sodium channels [253], although the composition of this venom remains to be determined.

Scorpion toxins provide a rich diversity of lead molecules for developing highly specific bioinsecticides, particularly those targeting insect ion channels [9,260]. Sodium channel toxins (NaTx), the major components of scorpion venoms, specifically target Na_v_ channels and cause insect paralysis or death. NaTx are long-chain scorpion toxins composed of 60 to 76 amino acid residues, most of which contain four disulfide bridges, although some contain three disulfide bridges [260]. Depending on the binding site and whether the toxin affects the opening or closing dynamics of Na_v_ channels, NaTx can be further classified into two categories: α-NaTx and β-NaTx. α-NaTx, predominantly found in New World scorpions, inhibit or delay of Na_v_ channel inactivation by binding to receptor site 3 (located in domain IV of the channel), whereas β-NaTx, primarily identified in Old World scorpions, modify the activation dynamics of these channels by binding to receptor site 4 (located in domain II of the channel) (reviewed in [9,99,261]. Based on their preference for mammalian or insect pharmacological targets, both α-NaTx and β-NaTx can be subdivided into three subgroups: classic α-NaTx and β-NaTx, insect α-toxins and β-toxins, and α-like toxins and β-like toxins [63,262,263].

Classic α-NaTx are highly toxic to mammals, whereas α-NaTx insectotoxins are specifically active against insect Na_v_ channels. A third group, the α-NaTx-like toxins, affects both mammalian and insect Na_v_ channels [63,99]. Although classic NaTx and insectotoxins differ in their primary structures, their three-dimensional folds are conserved. The selectivity of scorpion NaTx is likely determined by minor variations at the binding site in Na_v_ channels [9].

Scorpion β-NaTx exhibit diverse actions on mammalian and insect Na_v_ and are classified into four classes: (1) toxins with selective activity against mammalian Na_v_ channels, (2) selective, excitatory toxins targeting insect Na_v_ channels, (3) selective, depressant toxins targeting insect Na_v_ channels, and (4) toxins affecting both mammalian and insect Na_v_ channels [63]. Insect-selective excitatory β-NaTx are distinguished by a C-terminal α-helix and the conserved motif KKxGxxxDxxGKxxECx(4,9)YCxxxCTKVxYAxxGYCCxxxCYCxGLxDDKx(9)KxxCD (where ‘x’ represents any amino acid and the numbers in parentheses indicate the number of residues in that region) [9,63]. These toxins induce sustained muscle contraction. Conversely, insect-selective depressant β-NaTx, characterized by the motif DGY[IP][KR]x(2)[DNS]GC[KR]x[ADS]Cx(2,3)Nx(2,3)Cx(3)Cx(3)G[AG]x[FY]GYCW[AGT]WGLACWC[EQ][GN]LP[ADE] (where bracketed amino acids represent possible residues at that position) [63], lead to flaccid paralysis. Notably, certain insect-selective depressant β-NaTx, despite their high affinity for insect Na_v_ channels, are also active on rat Na_v_ channels [9].

AaIT, a 70-residue peptide with four disulfide linkages, was the first insect toxin identified in the venom of the scorpion *Androctonus australis*. Its excitatory nature and preference for insect targets are well documented [264,265,266]. Investigations into the cytotoxicity of this toxin using insect and human cell lines (Sf9 and MCF-7, respectively) highlighted a striking selectivity. The toxin was highly potent against insect cells (50% cytotoxicity at 0.13 μM), but showed no significant toxicity to human cells at 1.3 μM, suggesting potential applications where insect-specific toxicity is desired [267].

Valdez-Velázquez et al. [268] reported that chromatographic (RP-HPLC) fraction VII of *Centruroides tecomanus* venom was lethal to crickets and mice. Two components within this fraction, with molecular weights of 7013 Da and 7538 Da, were specifically toxic to crickets, but not to mice. More recently, two insect-specific toxins, Ct-IT1 and Ct-IT2, were characterized from venom of *C. tecomanus* and shown to be very active against crickets, even at low doses [269]. In contrast, they were non-toxic to mammals since high doses do not cause adverse effects in mice [258]. Ct-IT1, in particular, with an LD_50_ of 3.81 μg/100 mg, caused potent immediate paralysis in 80% of crickets injected with one LD_50_ and paralysis within 5 min in the remaining 20%; a lower dose of 0.8 μg/100 mg caused immediate paralysis in 50% and paralysis in the remaining 50% within 1 h. Structural analyses using homology modeling attributed this high toxicity to a “trapping apparatus” on the toxin’s surface that is thought to be crucial for its insecticidal activity, in a manner analogous to that described for LqhIT2, an insect-selective toxin from *L. quinquestriatus hebraeus* [270]. Other scorpion insectotoxins, including AaH IT5, BtITx3, and AaIT, have also been studied for their potential application in managing agricultural pests such as *Bemisia tabaci* (silverleaf whitefly) and *Helicoverpa armigera* (cotton bollworm) [165], *Heliothis virescens* (tobacco budworm) [271], *Spodoptera littoralis* [272] and *Nilaparvata lugens* (brown planthopper) [273].

Several insectotoxins have been identified in venoms of the scorpion genus *Tityus* that occurs in Central and South America. Ts1 (6879.4 Da) is a major component of *Tityus serrulatus* venom and contains the conserved motif GCK[FLV]xC[FV][IP][NR][NP][AES][EGS]x[CGN] characteristic of broad-spectrum β-NaTx, with residues Lys12, Trp39, and Trp54 playing a crucial role in the interaction of Ts1 with rat and cockroach synaptosomes [92]. C-terminal amidation is also essential for the toxin’s biological activity [274]. Ts1 exhibits a dual action on Na^+^ channels and affects both vertebrate and invertebrate systems. At a concentration of 100 nM, Ts1 functions as a typical β-NaTx by shifting the activation voltage of mammalian Na_v_1.2, Na_v_1.3, Na_v_1.4, and Na_v_1.6 channels to more negative potentials. Ts1 also reduces the peak Na^+^ current through Na_v_1.5 channels without affecting the channel’s activation or inactivation rates [275]. In *Drosophila melanogaster*, Ts1 irreversibly modifies the DmNa_v_1 channel, resulting in an enhanced and sustained Na^+^ current, along with a similar hyperpolarizing shift in voltage dependence [275]. This toxin displays potent insecticidal activity against fly larvae and binds to synaptic components in house flies and cockroaches. Given its properties, Ts1 may represent an intermediate between traditional β-NaTx and insect-specific toxins [92].

*Tityus serrulatus* venom contains other toxins with insecticidal properties [114]. Notably, Ts5, a neurotoxin exhibiting similarity to α-NaTx, counteracts the inactivation of DmNa_v_1, a Na^+^ channel found in *D. melanogaster*. The inhibitory effect of 1 μM Ts5 on DmNa_v_1 inactivation was more pronounced than its effect on other Na_v_ channels tested [120]. This venom also contains antarease, a metalloprotease. Recombinant antarease, expressed in *Escherichia coli*, induced paralysis at the neuromuscular junction of *D. melanogaster* larvae, most likely through a mechanism involving its metalloprotease activity and the cleavage of external substrates on the presynaptic membrane [223].

To1 (formerly Tc49b), a 7400 Da toxin isolated from *Tityus obscurus* venom, is a non-selective β-NaTx that shares 62.3% similarity in its amino acid sequence with Ts1 [276]. This toxin potently affects the Na^+^ channels of the German cockroach (*Blattella germanica*), specifically BgNa_v_1. At concentrations as low as 70 nM, To1 shifts the BgNa_v_1 activation potential towards more negative values, thereby enhancing the probability of channel opening [276].

*Tityus bahiensis* venom also contains insecticidal components [277]. Tb2-II has high toxicity in insects, with an LD_50_ of 40 ng per house fly, but is also toxic to mammals. Conversely, TbIT-I, while maintaining potent insecticidal activity (LD_50_ of 80 ng per house fly), has little effect on vertebrate tissues. The presence of arginine at position 10 in TbIT-I may contribute to its reduced mammalian toxicity, in contrast to the negative residues found at this position in α and β toxins. Both TbIT-I and Tb2-II are toxic to crickets and cockroaches, and share similarities with other non-selective *Tityus* β-NaTx toxins.

**Figure 5:**
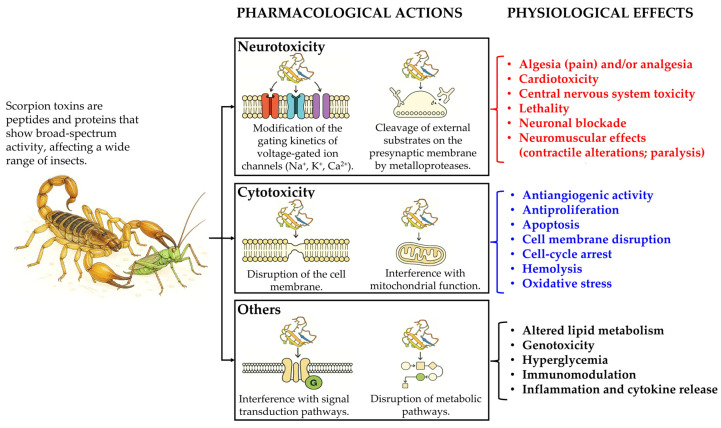
Primary actions of scorpion venoms and toxins in insects. Venom or toxin inoculation leads to a variety of effects, as indicated on the right, including manifestations of neurotoxicity (indicated in red) and cytotoxicity (indicated in blue). Note that the paralysis resulting from neurotoxicity may be either spastic paralysis caused by highly insect-selective excitatory β-NaTx such as AaHIT (*Androctonus australis hector*), Bj-xtrIT (*Hottenttota judaicus*), Lqh-xtrIT (*L. hebraeus*) and LqqIT1 (*L. quinquestriatus*), or flaccid paralysis caused by insect-selective depressant β-NaTx such as BaIT2 (*Buthacus arenicola*), BjIT2 (*H. judaicus*), BotIT2 (*B. tunetanus*) and LqhIT2 (*L. hebraeus*) [9]. Additional sites of action and effects (indicated in black) have been documented to varying degrees in vertebrates but much less studied in insects.

The venoms of non-Brazilian *Tityus* spp. also contain insectotoxins. The Panamanian *Tityus* spp., *T. asthenes* and *T. championi*, contain venom fractions capable of paralyzing and killing crickets (*Gryllus bimaculatus*) [278,279]. Two peptides, Tma2 and Tma3, isolated from the Colombian scorpion *Tityus macrochirus*, were shown to be lethal to *Acheta domesticus* crickets at a dose of 300 ng per cricket, while exhibiting no activity against human Na_v_1.1 to Na_v_1.7 channels; these peptides are structurally related to insect-specific Na^+^ channel toxins from *T. obscurus* [280]. Furthermore, bactridin-1, a peptide from *T. discrepans*, killed cockroaches (*Periplaneta americana*) within 48 h of injection, without harming mice [281]. In addition to these toxins, analyses of venoms from other *Tityus* spp. and other scorpion genera have identified numerous peptides with potential insecticidal properties, including in *T. costatus* [282], *T. cisandinus* [121], *Chactas reticulatus* (Chactidae), *Opisthacanthus elatus* (Hormuridae), *Centruroides edwardsii* (Buthidae) and *T. asthenes* (Buthidae) from Colombia [283] and *Tityus pachyurus* and *T. obscurus* from Colombia and the Brazilian Amazon, respectively [284]. Figure 5 summarizes the major effects of scorpion toxins in insects.

### 4.2. Practical Considerations Related to Scorpion Toxin-Based Insecticides

The potential of scorpion toxins to serve as lead molecules for novel insecticides raises important issues that need to be addressed during the development of an effective molecule, including matters related to selective toxicity, routes of toxin administration or delivery, product stability, and economic considerations such as large-scale production and costs, as discussed elsewhere for scorpion and spider insecticidal toxins [9,285,286,287,288].

Ideally, the toxin of interest should be highly insect-specific, or engineered to be insect-specific, with no adverse effect on vertebrates. As such, the insect-specific excitatory and depressant β-NaTx are particularly promising candidates as lead molecules [9,286], particularly given the greater structural similarity of voltage-gated Na^+^ channels (VGSC) among insects (≥87%) than between insects and humans (50–60%) [286]. However, even for insect-specific toxins, there is a need to tailor the specificity towards the target species so as not to adversely affect non-target beneficial species. Such targeted specificity needs to be addressed in order to overcome a major limitation of current chemical insecticides and pesticides, i.e., the indiscriminate deleterious effect they exert on non-target insects. In this regard, understanding the diversity of structural variations in voltage-gated Na^+^ channels (VGSC) among insects in general [286,289], and within target species, e.g., mosquitos (*Aedes albopictus*) [290], and how these can be tailored to increase potency is a fundamental step towards designing species-specific molecules. This is particularly important given the central role of VGSC structural diversity in the appearance of resistance to certain currently used pesticides, e.g., pyrethroids, in insects such as house flies (*Musca domestica*) [291], mosquitos (*Aedes* spp. [292,293]; *Anopheles* spp. [294]; *Culex* spp. [295]), buffalo lice (*Haematopinus tuberculatus*) [296], and fruit flies (*Drosophila* spp.) [297].

Adequate delivery of the molecule to the target species is fundamental for successful insecticidal activity, with the main possible routes of delivery being direct application through spraying, exposure to transgenic entomopathogens, and oral ingestion. Application by spraying is unlikely to be particularly efficient because of the difficulty that most peptide-based molecules have in penetrating the insect cuticle. This could possibly be overcome by engineering molecules with suitable cuticular permeability [298], or by coupling the toxin to nanoparticles to facilitate penetration [299,300,301]. A further difficulty with application by spraying is that it will not overcome a major limitation of the widespread spraying methods currently used in agricultural practice, namely, adverse effects on non-target species. Another issue related to application by spraying relates to the stability of the molecules to environmental conditions (exposure to heat, humidity and sunlight) that may have variable effects on molecular structure and biological activity. Studies with the insecticidal spider-venom peptides ω-Hv1a, ω/κ-Hv1a, Ta1a and Dc1a exposed to artificial sunlight for up to seven days revealed varying degrees of peptide degradation among the toxins, with 33–84% degradation after three days of continuous exposure; the main modifications involved oxidations, deamidations, and cysteine alterations [302]. Similar studies have yet to be performed for scorpion insecticidal toxins.

An alternative approach for toxin delivery to insects involves the use of infected entomopathogens, such as baculoviruses, fungi, e.g., *Metarhizium* and *Beauveria* spp., and the bacterium *Bacillus thuringiensis*, engineered to encode transgenes of the desired toxin that will later be expressed in the host insect [9,286]. Examples of this include the *Drosophila* X virus-like particle used to deliver the toxin AaIT (*Androctonus australis* Hector) to *Drosophila suzukii* [303], a fungus used to deliver this same toxin to malarial mosquitos [304], and toxin LqqIT1 expressed in the entomopathogenic fungus *Beauveria bassiana* (Balasmo) [305]. The presence of the scorpion toxins generally increases the lethality of these entomopathogens.

Since various scorpion toxins show oral toxicity in insects [9,306], oral ingestion can be a suitable route for toxin administration, although higher doses are required compared to direct injection into the body cavity. In the field, oral administration may be achieved by spraying plants with a toxin solution that would then be ingested by insects feeding on the plant parts or, alternatively, by creating transgenic plants to express the toxin of interest that would then be ingested by insects during feeding [9,286,287]. Scorpion toxin-containing transgenic plants have been produced for cotton (*Gossypium hirsutum*) [307], rapeseed (*Brassica napus*) [308], rice (*Oryza sativa*) [273] and tobacco (*Nicotiana tabacum*) [273,309] and show greater resistance to attack by pest insects. The extent of toxin absorption from the insect gut can vary considerably, but can be enhanced by fusing the toxin with a carrier protein such as *Galanthus nivalis* agglutinin (GNA) while retaining oral toxicity [9,286,287]. This approach has been applied to the insect toxins ButaIT (*Mesobuthus tamulus*) that is active against coleopteran, dipteran and lepidopteran pests [310,311], OdTx12, a β-excitatory toxin from *Odontobothus doriae* [312], and BjaIT (*Buthotus judaicus*) that is active against silkworms [313].

Other practical considerations for scorpion toxin-based pesticides include the need for large-scale industrial production of the toxin using expression systems such as *Pichia pastoris*, as described for the toxin LqhIT2 [314], determination of the ideal product formulation (as a liquid, or as a dry powder to be dissolved in appropriate solution immediately prior to use), packaging and distribution networks, product stability in the field (as commented above in relation to environmental conditions), the environmental safety of the product and how this will affect its mode of use, and the overall final cost of these insecticidal molecules to the user [286,287]. Clearly, while there is considerable potential for the use of scorpion toxins to develop novel insecticides, there are numerous aspects and stages that need to be addressed before the final product can be commercialized.

## 5. Therapeutic Applications of Scorpion Venoms and Toxins

**Figure 6:**
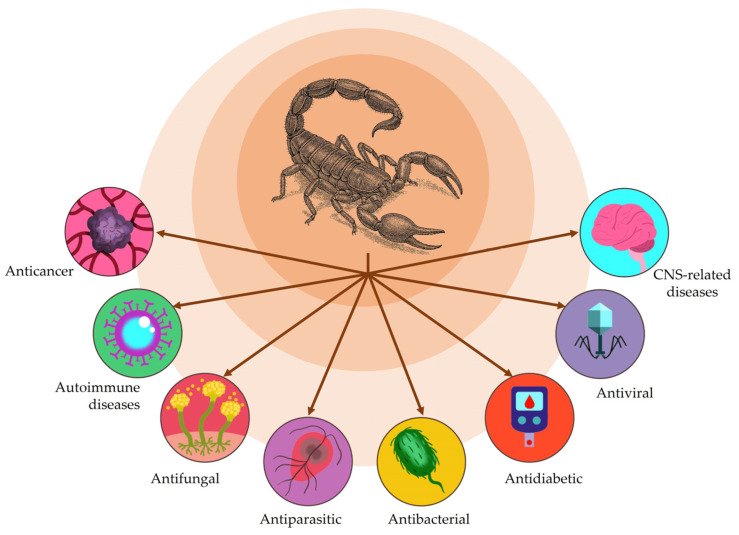
The therapeutic arsenal and potential of scorpion venom. This figure summarizes the multifaceted pharmacological potential of scorpion venoms, as discussed in the text.

Historically, scorpions and their venoms have been used in traditional medicine to address neurological disorders, rheumatism, and erectile dysfunction, with records dating back to the medieval era [315]. Biotechnological progress in recent decades has enabled the cloning, expression and synthesis of scorpion venom toxins for therapeutic applications. The therapeutic actions of these toxins often involve mechanisms distinct from conventional pharmaceuticals. Figure 6 summarizes the wide range of potential therapeutic applications of scorpion toxins.

### 5.1. Antibacterial Activity

Scorpion venoms from various genera, including *Androctonus*, *Buthus*, *Leiurus*, *Parabuthus*, *Scorpio*, and *Tityus*, exert antibacterial effects against Gram-positive and Gram-negative bacteria from a wide range of genera, including *Acinetobacter*, *Bacillus*, *Enterobacter*, *Enterococcus*, *Escherichia*, *Klebsiella*, *Micrococcus*, *Pseudomonas*, *Salmonella*, *Staphylococcus*, and *Streptococcus* (Table 3). This action is mediated primarily by DBP and NDBP that interact with cell membranes, with cationic peptides showing greater activity against Gram-negative bacteria, and hydrophobic peptides being more effective against Gram-positive bacteria [36,42,44,45,182,184].

The antibacterial mechanism of some amphipathic peptides involves insertion into bacterial lipid membranes, leading to electrostatic interactions and subsequent pore formation via concentration-dependent oligomerization, ultimately disrupting membrane integrity [328]. These peptides can also interfere with protein or DNA synthesis [318]. Notably, scorpions, like other arthropods, possess defensins that exert potent antibacterial activity through membrane permeabilization, particularly against Gram-positive bacteria. Scorpion defensins, found in hemolymph and venom [329], are characterized by an antiparallel β-sheet linked to an amphipathic α-helix and an extended N-terminal fragment via three disulfide bridges. They differ from other scorpion toxins in size, sequence, and biological activity [329,330].

### 5.2. Antiviral Activity

Antiviral activity has been described for several scorpion venoms, including scorpion species from the Middle East and North Africa (MENA) (Table 4 [331,332,333,334,335]). This antiviral activity may be mediated by cationic peptides and involves mechanisms such as the disruption of phospholipid membranes (specifically for enveloped viruses), a decrease in viral DNA and protein synthesis, modulation of cellular signaling pathways to inhibit viral replication, and interference with endosomal acidification, thereby preventing viral genome release [45,47]. The range of mechanisms involved in antiviral activity has led to the investigation of scorpion venoms as a source of compounds active against a broad spectrum of viral families [46,336].

### 5.3. Antifungal Activity

Fungal infections pose a significant challenge to global public health, and the limited availability of antifungal drugs underscores the importance of exploring novel natural sources [337]. Scorpion venoms provide such a source and have been shown to exert antifungal activity [45,338,339]. Scorpion venom peptides can impede fungal growth and induce cell lysis by interacting with the cell’s outer membrane [42], while others can target proteins in the fungal nuclear envelope, leading to the production of reactive oxygen species, ion efflux, and ATP depletion, ultimately resulting in apoptosis. Additional mechanisms include the disruption of membrane surface tension, the creation of pores to release intracellular ions, and interference with mitochondrial regulators [42]. Despite the potential for identifying novel compounds, research on the antifungal effects of scorpion venoms remains limited. Table 5 summarizes some studies that have investigated the antifungal activity of certain scorpion venoms. These compounds hold promise for pharmaceutical applications, either as direct natural drugs or as templates for the development of new antifungal agents.

### 5.4. Antiparasitic Activity

Given the challenges posed by parasitic outbreaks, especially in developing nations, where drug resistance and adverse effects are significant concerns, scorpion venoms offer a source of lead compounds for developing potentially novel antifungal agents. However, compared to the antibacterial and antiviral activities of scorpion venoms, considerably less is known of their antiparasitic activities. Most investigations have focused on the activities against species of *Echinococcus*, *Plasmodium*, and *Toxoplasma*, and have examined the ability to interfere with parasite growth and replication as a means of controlling their dissemination [341]. Table 6 summarizes selected examples of scorpion venoms that have shown antiparasitic activity.

### 5.5. Autoimmune Diseases

Scorpion venoms exert their toxic effects primarily by altering the function of excitable cells. This disruption of cellular communication involves the combined action of K^+^ channel inhibitors and Na^+^ channel activators, leading to cellular depolarization. However, non-excitable cells also possess ion channels that are similar or identical to those of excitable cells. Consequently, scorpion toxins can affect non-excitable cells if the targeted channels play a crucial role in their function. A notable example is T lymphocytes, the white blood cells responsible for cellular immune responses in humans [346]. The activation of cytotoxic T cells relies on a continuous influx of Ca^2+^ ions that necessitates a counterbalancing efflux of K^+^ ions to maintain the membrane potential. Different T cell subsets achieve this balance by increasing the expression of either voltage-gated or Ca^2+^-activated K^+^ channels (K_v_1.3 and K_Ca_3.1, respectively) [347]. Of particular interest are chronically activated effector memory T cells (T_EM_) that contribute to the tissue damage observed in various autoimmune diseases, including multiple sclerosis, rheumatoid arthritis, and type-I diabetes mellitus [348]. T_EM_ cells overexpress K_v_1.3 channels that are susceptible to blockade by specific blockers [349], whereas other T cell subsets are unaffected by these blockers as their membrane potential is primarily regulated by K_Ca_3.1 channels. In some cases, K_Ca_3.1 channels can compensate for dysfunctional or absent K_v_1.3 channels, although both types of channels are often required for complete normal cell functioning [347]. Given that several scorpion CSα/β toxins are high-affinity blockers of these specific K^+^ channels, these toxins can be promising candidates for developing drugs to treat autoimmune disorders.

### 5.6. Antidiabetic Activity

Several scorpion venoms, particularly from Middle Eastern and North African species, have been studied for their antidiabetic activity, but the number of such studies is small (Table 7). The findings so far suggest that only a few venom components exert any significant antidiabetic activity. The toxins implicated in these effects represent a promising source for the development of novel therapeutic agents, but further studies are necessary to fully characterize the venom components involved.

### 5.7. Anticancer Activity

Scorpion venoms are recognized as potential sources for anticancer drug development [353,354,355,356]. Scorpion venom peptides exhibit diverse anticancer mechanisms, including ion channel modulation, induction of apoptosis, and membrane disruption (Table 8 [357,358,359,360,361,362,363,364,365,366,367,368,369,370,371,372,373,374,375,376,377,378,379,380,381,382,383,384]). Peptides that target ion channels and signaling pathways, such as chlorotoxin and cationic antimicrobial peptides [382], are of particular interest because of their selective toxicity and ability to disrupt cancer cell homeostasis. For example, meuCl14 (from the venom of *Mesobuthus eupeus*) [167] and Smp43 (from the venom of *Scorpio maurus palmatus*) [383] efficiently inhibit tumor proliferation and migration by targeting tumor-associated chloride channels and inducing mitochondrial dysfunction. Additionally, phospholipase A_2_, such as leptulipin, can influence apoptotic pathways and cell cycle arrest [368]. These findings indicate that scorpion venom peptides and proteins provide promising avenues for innovative cancer therapies.

### 5.8. Analgesic Activity

Pain is a frequent finding in clinical envenoming by scorpions [385,386,387,388], with Na_v_ channel activation by venom peptides being the major mechanism involved [20,52,53,389,390], although K^+^ channel inhibition [53,389,390], TRPV1 channel activation [390,391,392], and the release of pro-inflammatory mediators, including cytokines (e.g., IFN-γ, IL-1β, TNF-α) [392,393] also contribute to this phenomenon.

Paradoxically, scorpion venoms also contain peptides with analgesic activity, although fewer such molecules have been identified and characterized pharmacologically compared to pain-inducing toxins [19,37,47,84,390,394,395,396]. The vast majority of these peptides act by blocking Na^+^ channels, although the blockade of selected Ca^2+^ channels, e.g., N-type Ca^2+^ currents and T-type Ca_v_3.2 and Ca_v_3.3 [397], activation of K^+^ channels, e.g., Kv1.1 and Kv1.3 channels by hetlaxin from *Heterometrus laoticus* [398], indirect [399] and direct (by interaction with opioid receptors) [400,401] activation of the endogenous opioid system, the inhibition of inflammatory pathways and alterations in neurotransmitter release may also be involved [20,69,394,395]. Many of the analgesic peptides identified so far (>20) are from the venom of *Buthus* (*Mesobuthus*) *mertensii* Karsch, a species widely used in Chinese traditional medicine [20,37,47,84,390,394,395,396,402,403], although molecules from other genera (*Androctonus*, *Hemiscorpius*, *Leiurus*, *Tityus*) have also been characterized. Table 9 [404,405,406,407,408,409,410,411,412,413,414,415,416,417,418,419] summarizes the characteristics of several of these analgesic peptides. It should be noted that the blockade of Na^+^ channels by a given toxin may involve several channel subtypes, e.g., Na_v_1.3, Na_v_1.4, Na_v_1.5, Na_v_1.7 and Na_v_1.8 for toxins such as BmK AGAP, BmK AS and BmK M9, or may be selective for a specific subtype, e.g., Na_v_1.7 for toxins DKK-SP2, BmKBTx and BmNaL-3SS2, and Na_v_1.8 for Syb-prII (all toxins from *B. mertensii* venom).

Analgesic peptides are of particular interest for developing new compounds for the clinical treatment of pain [20,47,390,395,396,402], and the fact that various of these peptides are insectotoxins devoid of toxic effects in vertebrates (mammals) makes them particularly amenable for use as lead molecules for therapeutically useful analgesics [390]. In addition to the classic approach of identifying analgesic molecules and then proceeding with structure-activity analyses to design novel therapeutic compounds, other approaches that have been used include site-directed mutagenesis to identify important amino acids and ‘core’ or minimal structures necessary for analgesic activity, as done for Bmk AGAP [420], the use of computational methods and molecular biology to produce chimeric analgesic molecules from non-toxic and toxic peptides, e.g., a combination of the non-toxic β-neurotoxin CeIIB from *Centruroides elegans* and the toxic β-neurotoxin CssII from *Centruroides suffusus suffuses* [421], and the use of machine learning (artificial intelligence) to identify potential analgesic molecules starting with non-toxic peptides such as defensin 4 from *B. martensii* Karsch [401].

### 5.9. Therapeutic Potential of Scorpion Toxins in Neurological and Neurodegenerative Diseases

The high specificity and stability of scorpion toxins make them promising lead compounds for the development of therapies for a range of central nervous system (CNS) disorders. For example, peptides targeting voltage-gated Ca^2+^, K^+^ and Na^+^ channels are of interest for treating epilepsy, with engineered scorpion toxins acting as selective channel blockers or modulators to dampen hyperexcitability [422]. Similarly, the scorpion venom peptide chlorotoxin (ClTx) selectively binds glioma and glial cells (see Section 3.3.3 above), and modified analogs are being explored for imaging and therapeutic purposes in neuroinflammatory diseases like multiple sclerosis [423].

**Figure 7:**
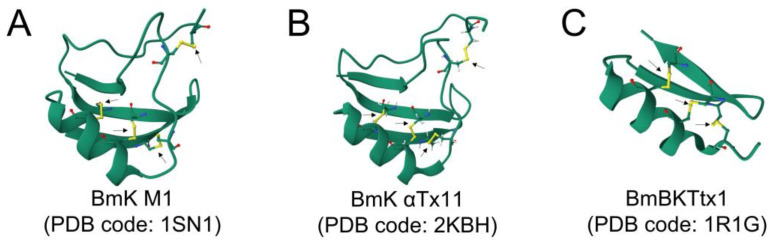
Structures of three *B. martensii* Karsch toxins. (**A**) BmK M1 (also known as BmK 1), a long-chain cardiotoxic Na^+^ channel blocker and one of the most abundant and best characterized long-chain toxins of this venom, showing the disulfide bridges (indicated in yellow and by arrows) at Cys12–63, 16–36, 22–46 and 26–48. (**B**) BmK αTx11, an α-toxin (Na^+^ channel blocker) homolog, showing the disulfide bridges at Cys12–63, 16–36, 22–46 and 22–48. (**C**) BmBKTtx1, a short-chain Ca^2+^-activated K^+^ channel (BK_Ca_) blocker, showing the disulfide bridges at Cys3–22, 8–27 and 12–29. The structural models shown here are based on experimentally determined sequences. References [66,84,402,403] provide detailed discussion of *B. martensii* Karsch toxins, including those indicated here.

Neurodegenerative diseases such as Parkinson’s disease and Alzheimer’s disease involve complex mechanisms, including neuroinflammation, oxidative stress, mitochondrial dysfunction, and protein aggregation. The venoms of some scorpion species, e.g., *A. australis* and *B. martensii*, contain peptides that have neuroprotective, anti-inflammatory, and antioxidant properties, and offer promise as novel molecules for disease-modifying therapies. Figure 7 illustrates structural aspects of three *B. martensii* Karsch toxins. Table 10 summarizes the therapeutic potential of selected scorpion toxins for neurodegenerative diseases.

## 6. Challenges and Future Perspectives

As discussed above, scorpion venoms and their toxins have been investigated with regard to their potential biotechnological use as pesticides in agriculture and as therapeutic agents for treating a variety of medical conditions.

The insecticidal activities of scorpion venom peptides hold considerable promise for developing novel pesticides. However, the application of these toxins faces several challenges that need to be addressed to ensure their safe and sustainable use. A significant concern regarding scorpion toxin-based insecticides is their potential toxicity to non-target organisms. While some toxins exhibit high specificity towards target insects, others may pose risks to beneficial insects, such as pollinators and predators. Pollinators, like bees, are crucial for agricultural productivity and ecosystem health. The indiscriminate use of scorpion toxins could negatively impact these organisms, leading to ecological imbalances. Studies are needed to thoroughly assess the toxicity of specific scorpion toxins to a wide range of non-target organisms and to develop strategies for minimizing off-target effects [429].

The environmental impact of scorpion toxin-based pest control strategies is another crucial consideration. The production and application of these toxins must be sustainable to avoid adverse effects on ecosystems. This includes minimizing the use of solvents and other chemicals during toxin extraction and purification, as well as developing environmentally friendly delivery systems. Furthermore, the long-term effects of scorpion toxins on soil microorganisms and other environmental components need to be investigated. The use of genetically modified microorganisms for toxin production or the synthesis of toxin mimics might represent a more sustainable alternative for obtaining pure toxin compared to conventional toxin purification procedures [62].

To overcome these challenges and maximize the potential of scorpion toxins, future research should focus on several key areas:

- Mechanistic studies: A deeper understanding of the molecular mechanisms of scorpion toxin action in insects is essential for optimizing their insecticidal activity. This includes identifying specific target sites within insect neuronal channels and elucidating the interactions between toxins and these targets. Techniques such as electrophysiology, molecular modeling, and proteomics can be useful in reaching this goal. This should lead to the development of more specific toxins.
- Structure-activity relationship studies: Research on the structure-activity relationships of scorpion toxins is crucial for designing more potent and selective insecticides. By identifying the structural features responsible for insecticidal activity, it should be possible to develop modified toxins with enhanced efficacy and reduced off-target effects. This can be achieved through peptide synthesis and combinatorial chemistry.
- Development of novel delivery systems: Efficient and targeted delivery of scorpion toxin-based insecticides is essential for maximizing their efficacy and minimizing environmental impact. Novel delivery systems, such as nanoencapsulation, liposomes, and viral vectors, can be explored to achieve this goal. These systems can enhance toxin stability, improve target specificity, and reduce the amount of toxin required for pest control [264].

## 7. Conclusions

Scorpion venoms contain a complex mixture of peptides, enzymes, and other compounds that interact with various biological targets, including ion channels and receptors. These interactions underlie the potent insecticidal and therapeutic effects observed with scorpion venom components. Specifically, insectotoxins, primarily NaTxs and KTxs, have shown promise as novel bioinsecticides because of their high specificity and potency against insect pests. In addition, calcins and other peptides have potentially interesting therapeutic applications, including as carrier molecules for drug delivery. The potential of scorpion venoms and toxins as a source for developing novel insecticides and other biotechnological applications is substantial. The high specificity and potency of insectotoxins make them attractive candidates for targeted pest control strategies, offering an environmentally friendly alternative to synthetic pesticides. Furthermore, the diverse pharmacological properties of scorpion venom components suggest their potential for developing novel therapeutics for various diseases. Advances in biotechnology, including peptide synthesis and recombinant DNA technology, have facilitated the synthesis of scorpion toxins and derivatives, as well as the development of venom-derived therapeutics. Further research is warranted to explore the mechanisms of action of scorpion toxins and to optimize their applications in agriculture and medicine. This includes investigating the structure–activity relationships of scorpion toxins, developing novel delivery systems for sustainable application, and conducting clinical trials to assess the safety, potential risks, and efficacy of venom-derived therapeutics.

## Figures and Tables

**Table 3:** Antibacterial activities of some scorpion venoms and toxins.

Species	Origin	Molecule(s)	Nature	Activity	References
*Aegaeobuthus* *gibbosus*	Turkey	Venom	Venom	Venom was active against *Bacillus sphaericus*, *Bacillus subtilis*, *Bacillus cereus* and *Staphylococcus aureus* (all Gram-positive) and *Escherichia coli* (Gram-negative), but not against *Enterococcus faecalis* and *Micrococcus luteus* (both Gram-positive)	[316]
*Androctonus* *aeneas*	North Africa	AaeAP1, AaeAP2	Antimicrobial peptides	Both peptides inhibited *S. aureus* (MIC: 16 μg/mL), but were much less effective against *E. coli* (MIC: >512 μg/mL).	[317]
*Androctonus* *amoreuxi*	North Africa	AamAP1, AamAP2	Antimicrobial peptides	AamP1 and AamAP2 inhibited *S. aureus* (MIC: 20 and 48 μM, respectively) more effectively than *E. coli* (MIC: 120 and 150 μM, respectively).	[318]
		GK-19 (AamAP1 derivative)	Antimicrobial peptide	Broad-spectrum antibacterial activity against *E. coli*, *E. faecalis*, *K. pneumoniae*, *P. aeruginosa* and *S. aureus* (MIC: 3–10 μM). Disrupted bacterial membranes.	[319]
*Androctonus* *australis*	Algeria	Venom and peptide G-TI	Sodium channel inhibitor	Venom was active against *B. cereus*, *E. coli*, *Microcossus* spp. and *S. aureus* (MIC: 75, 150, 125, and 125 μg/mL, respectively). Disrupted bacterial membranes, leading to cell death. The peptide G-TI was active against *B. cereus*.	[320]
*Androctonus* *crassicauda*	Saudi Arabia	Venom	Venom	Inhibited the growth of *E. coli*, *Salmonella* spp., *S. aureus,* and *Paenibacillus larvae.*	[321]
	Turkey	Venom	Venom	Venom was active against *Bacillus cereus*, *Bacillus sphaericus*, *Bacillus subtilis*, *Escherichia coli*, and *Micrococcus luteus*, but not against *Enterococcus faecalis* and *Staphylococcus aureus.*	[316]
*Hottentotta* *saulcyi*	Turkey	Venom	Venom	Venom was active against *Bacillus cereus*, *Bacillus sphaericus*, *Bacillus subtilis*, *Enterococcus faecalis* and *Staphylococcus aureus*, but not against *Escherichia coli* or *Micrococcus luteus.*	[316]
*Leiurus* *abdullahbayrami*	Turkey	Venom	Venom	Venom was active against *Bacillus sphaericus*, but not against *Bacillus cereus*, *Bacillus subtilis*, *Enterococcus faecalis*, *Escherichia coli*, *Micrococcus luteus* or *Staphylococcus aureus*.	[316]
*Leiurus* *quinquestriatus*	Egypt	Venom	Venom	Inhibited *B. subtilis* and *C. freundii*; no effect on *B. cereus* and *K. pneumoniae*.	[322]
	Saudi Arabia	Venom	Venom	Inhibited the growth of *E. coli*, *Salmonella* spp., *S. aureus*, and *Paenibacillus larvae.* More effective than *A. crassicauda* venom	[321]
	Saudi Arabia	Venom	Venom	Concentration-dependent inhibition of *A. baumannii*, *E. coli*, *E. faecalis*, *K. pneumoniae, P. aeruginosa*, and *S. aureus.*	[323]
*Odontobuthus doriae*	Iran	Peptide 3	Venom fraction	Inhibited *Enterococcus faecalis* and *Escherichia coli* UT189 (IC_50_: 160 and 80 μg/mL, respectively).	[324]
*Pandinus* *imperator*	North Africa	Scorpine	Antimicrobial peptide	Inhibited *B. subtilis* and *K. pneumoniae* (MIC: 1 and 10 μM, respectively).	[325]
*Protoiurus* *kraepelini*	Turkey	Venom	Venom	High bactericidal activity, with inhibition zones of 9–20 mm against *Bacillus sphaericus*, *Bacillus cereus*, *Bacillus subtilis*, *Enterococcus faecalis*, *Escherichia coli*, *Micrococcus luteus* and *Staphylococcus aureus*.	[316]
*Scorpio* *maurus* *palmatus*	Egypt	Smp24, Smp43	Antimicrobial peptides	Disrupted bacterial membranes, with activity against *Bacillus subtilis*, *Escherichia coli*, *Klebsiella pneumoniae*, *Pseudomonas aeruginosa*, *Staphylococcus aureus* and *Staphylococcus epidermidis*. Smp43 also interfered with *B. subtilis* DNA synthesis. Caused pore formation and induced oxidative stress in *E. coli* (MIC: 4–128 μg/mL). Greater efficacy against Gram-positive bacteria.	[326,327]

MIC: Minimum inhibitory concentration.

**Table 4:** Antiviral activities of some scorpion venoms and toxins.

Species	Origin	Molecule(s)	Nature	Activity	References
*Buthus occitanus tunetanus*	Tunisia	BotCl	Chlorotoxin-like peptide	Concentration-dependent inhibition of Newcastle disease virus in avian species, with an IC_50_ of 0.69 μM. Mechanism involves direct disruption of viral particle structure, preventing cellular entry and proliferation.	[331]
*Mesobuthus eupeus*	Iran	Mucin 13, Mucin 18	Antimicrobial peptides	Potential antiviral activity against SARS-CoV-2 by targeting the receptor-binding domain (RBD) of the spike protein. Mucin-18 shows stronger binding affinity, with the A9T mutation enhancing its effectiveness.	[332]
*Odontobuthus doriae*	Iran	ODAMP2, ODAMP5	Antimicrobial peptides	Computational studies suggest high binding affinities to the SARS-CoV-2 spike protein’s receptor binding domain, with binding energies of −59.2 and −51.8 kcal/mol for ODAMP2 and ODAMP5, respectively, indicating potential efficacy.	[333]
*Scorpio maurus* *palmatus*	Egypt	Venom	Venom	Significant activity against hepatitis C virus (HCV), interfering with viral entry. IC_50_ of 6.3 μg/mL, selectivity index of 15.8. Activity is independent of enzymatic processes and specific to Flaviviridae viruses such as HCV and Dengue virus (DENV).	[334]
	Egypt	Smp76	Scorpine-like peptide	Potent antiviral activity against HCV and DENV, inhibiting early infection stages, likely Via direct viral particle interaction. IC_50_ of 0.01 μg/mL for both viruses. No cytotoxic or hemolytic effects at concentrations exceeding 1000 times the antiviral dose.	[335]

**Table 5:** Antifungal activities of some scorpion venoms and toxins.

Species	Origin	Molecule(s)	Nature	Activity	References
*Androctonus* *aeneas*	North Africa	AaeAP1, AaeAP2	Antimicrobial peptides	Inhibits *Candida albicans* (MIC: 32 μg/mL).	[317]
*Androctonus* *amoreuxi*	North Africa	GK-19	Derived from AamAP1	Shows strong antifungal activity against *Candida albicans*, *Candida glabrata* and *Candida krusei* (MIC: 5–10 µM). Disrupts fungal membranes, causing structural damage.	[319]
*Androctonus* *australis*	North Africa	AamAP1, AamP2	Antimicrobial peptides	Inhibits *Candida albicans* (MIC: 64 µM).	[42]
	North Africa	Androctonin	Cysteine-rich antimicrobial peptide	Exhibits potent antifungal activity against various fungal species, particularly *Verticillium torelis* and *Fusarium oxysporum* (MIC: <4 µM). Completely inhibits *Neurospora crassa* spore growth at ≥12 µM, without regrowth.	[340]
*Leiurus* *quinquestriatus*	Saudi Arabia	Venom	Venom	Reduces growth and survival of *Candida albicans* (by 31.2%) and *Candida glabrata* (by 39.0%).	[323]

MIC: Minimum inhibitory concentration.

**Table 6:** Antiparasitic activities of some scorpion venoms, venom fractions and toxins.

Species	Origin	Molecule(s)	Nature	Activity	References
*Androctonus* *crassicauda*	Egypt	Venom	Venom	Antihelminthic activity against *Trichuris arvicolae* that involved significant ultrastructural changes. Potential use in the treatment of gastrointestinal nematodes resistant to conventional drugs.	[342]
	Saudi Arabia	Venom	Venom	Complete destruction of *Echinococcus granulosus* protoscolices after 4 h of exposure at 100 μg/mL. The damage involved apoptosis and structural alterations. Potential use in the non-surgical treatment for hydatidosis, a significant public health problem.	[343]
*Hemiscorpius* *lepturus*	Iran	Fraction 5	Venom peptide fraction (<10 kDa)	Reduced the viability of *Toxoplasma gondii* tachyzoites at 100 μg after 2 h.	[341]
*Mesobuthus eupeus*	Iran	Mucin 24, Mucin 25	Antimicrobial peptides	Inhibited *Plasmodium falciparum* without harming human cells (erythrocytes and GC-2 cells), and prevented *P. berghei* ookinete growth at 10–20 μM, possibly via membrane disruption.	[344]
*Mesobuthus eupeus*	Iran	Fraction 8	Venom peptide fraction (<10 kDa)	Scolicidal activity against *Echinococcus granulosus* protoscolices within 30 min of exposure to venom fraction.	[345]
*Pandinus* *imperator*	North Africa	Scorpine	Antimicrobial peptide	Activity against *Plasmodium berghei*, involving disruption of sexual stage development in mosquito midguts. Inhibition of fertilization and ookinete formation with ED_50_ of 10 μM and 0.7 μM, respectively, an effect attributed to membrane disruption.	[325]

**Table 7:** Antidiabetic activities of some scorpion venoms and venom fractions.

Species	Origin	Molecule(s)	Activity	References
*Androctonus australis* *hector*	Algeria	Venom Fraction F1	Treatment with Fraction F1 (10 mg/kg, i.p.; daily for 11 days) restored the body weight gain, attenuated the diabetes-induced hyperglycemia and improved the glucose tolerance in mice with streptozotocin-induced diabetes. Fraction F1 improved β-cell function and survival and increased the mitotic activity of these cells. Characterization of Fraction F1 revealed the presence of hyaluronidase and peptides, potentially contributing to glucose homeostasis and β-cell survival.	[350]
*Leiurus* *quinquestriatus*	Egypt	Venom	Venom (daily injection of 1/10 of the lethal dose for eight weeks) normalized the blood glucose levels and body weight in rats with streptozotocin-induced diabetes. The venom prevented the histopathological and immunohistochemical changes caused by diabetes in splenic tissues.	[351]
	Egypt	Venom	Venom (daily injection of 1/40 of the sublethal dose for eight weeks) prevented body weight loss, normalized hematological parameters, blood cell counts and blood glucose levels, reduced C-peptide levels to slightly below normal, normalized indicators of hepatic function and indicators of oxidative stress, and promoted β islets regeneration in rats with streptozotocin-induced diabetes.	[352]

**Table 8:** Anticancer activities of some scorpion venoms and toxins.

Species	Origin	Molecule(s)	Nature	Activity	References
*Androctonus australis*	Tunisia	P01	K^+^ channel toxin	Inhibits proliferation, adhesion, and migration of U87 glioblastoma cells.	[357]
	Tunisia	AaTs-1	Tetrapeptide	Inhibits glioblastoma U87 cell proliferation, modulates kinase expression, and enhances p53 and FPRL-1 expression.	[358]
	Tunisia	AaTs-1-4B	Dendrimer multi-branched molecules	Inhibits U87 cell proliferation and migration, enhances ERK1/2 and AKT phosphorylation, and increases p53 expression.	[359]
*Androctonus australis hector*	Algeria	F3 fraction	Venom fraction	Induces apoptosis in lung cancer cells (NCI-H358) via the mitochondrial pathway.	[360]
	Algeria	Venom	Venom	Alters alveolar epithelial cell (A549) integrity, disrupts cytoskeleton, and reduces cell migration.	[361]
*Androctonus* *crassicauda*	Turkey	Acra3	Na^+^ channel toxin	Cytotoxic to BC3H1 cells and induces necrosis.	[362]
*Androctonus* *crassicauda*, *Androctonus bicolor*, *Leiurus* *quinquestriatus*	Saudi Arabia	Venom	Venom	Inhibits cell proliferation, motility, and colony formation in colorectal and breast cancer cells.	[363,364]
*Buthus occitanus*	Morocco	α-Insect toxin Lqq3, α-Like toxin Bom4	Na^+^ channel toxin	Inhibits hepatocellular carcinoma cell proliferation (Huh 7.5 cells in 3D culture).	[365]
*Buthus occitanus tunetanus*	Tunisia	RK1	Short peptide (14 amino acids)	Inhibits cell proliferation, migration, and angiogenesis in U87 and IGR39 cells.	[366]
*Euscorpius* *mingrelicus*	Turkey	Venom	Venom	Cytotoxicity towards breast and lung cancer cells by inducing apoptosis and necrosis.	[367]
*Hemiscorpius lepturus*	Iran	Leptulipin	Phospholipase A_2_	Inhibits proliferation, alters morphology, induces DNA fragmentation, and cell cycle arrest in HT-29 and MDA-MB-231 cells.	[368]
*Hottentotta saulcyi*	Iran	Venom	Venom	Induces apoptosis in MCF-7 cells, reduces tumor density in vivo, and upregulates pro-apoptotic genes.	[369]
*Hottentotta Schach*	Iran	Venom	Venom	Antiproliferative activity in MCF-7 cells by inducing oxidative stress leading to apoptosis.	[370]
*Leiurus* *quinquestriatus*	Egypt	GNPs-V	Venom conjugated with gold nanoparticles	Anticancer activity against liver cancer cell lines by inhibiting migration, inducing cell cycle arrest, and promoting apoptosis.	[371]
	Israel	Chlorotoxin	Cl^−^ channel toxin	Targets glioblastoma multiform cells, inhibits angiogenesis, and binds to specific channels.	[372]
	Saudi Arabia	FLV-SV	Fluvastatin-scorpion venom peptide nano-conjugate	Antiproliferative activity in colorectal adenocarcinoma cells.	[373]
	Saudi Arabia	THQ–PL–SV	Thymoquinone-phospholipon-scorpion venom peptide nanovesicles	Antiproliferative activity in lung cancer cells through modulation of gene expression.	[374]
*Mesobuthus eupeus*	Iran	meuCl14	Chlorotoxin	Inhibits hMMP-2 and limits tumor progression.	[167]
	Iran	MeICT	Cl^−^ channel toxin	Inhibits glioma cell proliferation and migration; downregulates Annexin A2 and FOXM1.	[375]
*Odontobuthus* *bidentatus*	Iran	Venom	Venom	Induces apoptosis in HepG2 cells via increased nitric oxide levels. Antiproliferative activity in MCF-7 cells.	[376,377]
*Odontobuthus doriae*	Iran	Venom	Venom	Apoptotic and antiproliferative activity in human neuroblastoma (SH-SY5Y) and human breast cancer (MCF-7) cells	[378,379]
*Protoiurus kraepelini*	Turkey	Venom	Venom	Concentration-dependent cytotoxicity in Jurkat cells.	[380]
*Scorpio maurus* *palmatus*	Egypt	Smp24	Cationic antimicrobial peptide	Suppresses lung cancer cell growth, and induces apoptosis, cell cycle arrest, and autophagy in HepG2 cells.	[381,382]
	Egypt	Smp43	Cationic antimicrobial peptide	Inhibits lung cancer cell proliferation, causes membrane rupture and mitochondrial dysfunction, and alters apoptosis, cell cycle, and autophagy. Inhibits hepatocellular carcinoma cells.	[383,384]

**Table 9:** Analgesic activities of some scorpion venoms and toxins.

Species	Origin	Molecule(s)	Toxin Class	Activity	References
*Androctonus* *amoreuxi*	Egypt	Venom	-	Dose-dependent reduction in acetic acid-induced writhing (peripheral pain) and increased latency in the tail flick test (central pain).	[404]
*Androctonus* *mauretanicus* *mauretanicus*	Morocco	anatoxin Amm VIII	NaTx	Dose-dependent analgesia in hot plate and tail flick tests. Antinociception mediated by blockade of Na_v_1.2, activation of endogenous opioid system, and activation of mechanisms involving diffuse noxious inhibitory controls (DNIC).	[399,405]
*Buthus* *occitanus* *tunetanus*	Tunisia	BotAF	NaTx	Non-toxic β-toxin-like peptide with no effect on motor activity. Reversible low blockade (≤20%) of Na^+^ channels. Peripheral or spinal mechanisms involved in attenuating the pain associated with the acetic acid writhing, formalin, hot plate, and tail-flick assays. Analgesic activity independent of opioid system. More potent than β-endorphin or morphine in these tests. Effective when given i.p., but not when given i.v. or i.c.v. Low activity on TTX-S Na^+^ channels of DRG and does not bind to rat brain synaptosomes. Stimulates lumbar spinal cord c-fos/c-jun mRNA up regulation.	[406]
*Hemiscorpius* *lepturus*	Iran	Leptucin	ND	Analgesic effect against acute thermal pain (hot plate and tail-flick tests) at doses of 0.32 and 0.64 mg/kg, i.p., with similar or greater activity than morphine. No cytotoxicity or hemolysis. No histopathological alterations in heart, kidney or liver. LD_50_ (mice) > 4 mg/kg.	[407]
*Heterometrus* *laoticus*	Vietnam	Venom	KTx	Venom showed analgesic activity (9.5 and 19 mg/kg) in the tail immersion (55 °C water) and tail flick assays that was less potent than morphine (5 mg/kg). A short K^+^ channel toxin (Hetlaxin) that blocks K_v_1.1 and K_v_1.3, with high affinity for the latter (K_i_ = 59 nM), was isolated from the venom, but its analgesic activity in the assays indicated above was not tested.	[398]
*Leiurus q.* *quinquestriatus*	Sudan	LqqIT2	NaTx	Dose-dependent analgesia in hot plate and tail flick tests. Antinociception mediated by blockade of Nav1.2, activation of endogenous opioid system, and activation of mechanisms involving diffuse noxious inhibitory controls (DNIC).	[398]
*Mesobuthus* *martensii*	China	ANEP (Anti-neuroexcitation peptide)	NaTx	β-Toxin that blocks Na_v_1.7. Analgesic activity in the mouse acetic acid writhing test and hot plate test. Recombinant peptide has same activity as native peptide. Several mutants showed greater activity than the recombinant peptide.	[408,409]
	China	BmK-YA 8	Short-chain NDBP	Structurally related to enkephalin. Activates μ, κ and δ opioid receptors, with selectivity for δ opioid receptors ~7- and 12-fold greater than for µ and κ receptors, respectively. Full agonist at δ opioid receptors, and partial agonist with lower efficacy and potency on μ and κ receptors. Activity at δ opioid receptors antagonized by naloxone.	[400]
		BmK AGAP (Analgesic-antitumor peptide)	NaTx	Blockade of Na_v_1.4, Na_v_1.5, Na_v_1.7, Na_v_1.8, TRPV1, and KCNQ2/3 currents. Analgesic activity in the writhing, hot-plate and formalin tests. Attenuation of pain in the formalin-induced spontaneous nociceptive behavior assay involved inhibition of the expression of peripheral and spinal mitogen-activated protein kinases (MAPK), including p-p38, p-ERK, p-JNK and spinal Fos. Intrathecal administration of AGAP inhibited and reversed pain from chronic constrictive injury (CCI) of the sciatic nerve, from thermal hyperalgesia, and mechanical allodynia. Potentiated the analgesic effect of lidocaine.	[410,411,412,413]
	China	BmK AS	NaTx	Blockade of TTX-R (Na_v_1.8, 1.9) and TTX-S (Na_v_1.3), with no effect on voltage-dependent I_K_ and KCl or caffeine-induced Ca^2+^ influx in neurons. Decreased the number of action potentials in DRG neurons by ~50% at 0.5 μM. Analgesic activity in formalin and carrageenan nociceptive assays.	[414]
	China	BmK AngM1	NaTx	Little or no toxicity at doses up to 50 mg/kg, i.v. Irreversible (by washing) blockade of Na_v_ and delayed rectifier K^+^ (I_K_) currents, but no effect on transient K^+^ currents (I_A_) in rat hippocampal pyramidal neurons. Analgesic effect in acetic acid writhing assay in mice (63% inhibition at 0.8 mg/kg, compared to 83% with morphine 0.2 mg/kg, i.v.).	[415]
	China	DKK-SP2	NaTx	Blocked Na_v_1.7 channels expressed in Chinese hamster ovary cells and reduced the expression of this channel in trigeminal neurons. Reduced writhing in the acetic acid test in mice and pain associated with chronic constriction injury of the infraorbital nerve in rats was also attenuated. At the highest doses tested, the potency in both tests was similar to or better than morphine.	[416]
	China	BmKBTx, BmNaL-3SS2	NaTx	Blockade of Na_v_1.7. BmKBTx and BmNal-3SS2 reducing writhing in the acetic acid test in mice and show similar potency to morphine (reductions of 43% and 63% at 1 mg/kg i.p., respectively, compared to 49% with morphine 1.5 mg/kg i.p.).	[417]
	China	Syb-prII	NaTx	β-Neurotoxin that blocks Na_v_1.8, but not Na_v_1.9. Attenuated pain associated with chronic constriction injury of the infraorbital nerve, probably by attenuating MAPK-activated pathways. Efficacy similar to morphine.	[418]
*T* *ityus serrulatus*	Brazil	TsNTxP	NaTx	Non-toxic peptide structurally related to toxins TsVII (Ts1 or toxin-γ) and Ts3 (TsIV or tityustoxin). Non-toxic to mammals. Antinociceptive effect in tail-flick thermal test and intraplantar capsaisin injection. Attenuates neuropathic pain caused by constriction injury to sciatic nerve and paclitaxel administration. Caused reduced glutamate release from mouse spinal cord synaptosomes.	[419]

DRG—dorsal root ganglion, i.c.v.—intracerebroventricular, i.p.—intraperitoneal, i.v.—intravenous, KTx—potassium channel toxin, NaTx—sodium channel toxin, ND—not determined, NDBP—non-disulfide bridged peptide, TTX-S—tetrodotoxin-sensitive channel.

**Table 10:** Characteristics of some scorpion toxins with therapeutic potential in neurodegenerative diseases.

Species	Toxin	Toxin Characteristics	Therapeutic Potential
*Androctonus* *australis*	AaTx	One of a family of peptides that primarily targets Na^+^ channels	Shows immunomodulatory activity, including specific targeting of K_v_1.3 channels involved in T cell-mediated autoimmune responses [424]. As oxidative stress is a major contributor to dopaminergic neuron loss in Parkinson´s disease and amyloid pathology in Alzheimer´s disease, AaTx could represent a potentially useful lead compound for developing therapeutic drugs for these neurodegenerative diseases.
*B. martensii* Karsch	BmK AngP1	A 62-amino acid peptide with high sequence homology to neurotoxins that target ion channels, but it exerts non-toxic and neurotrophic effects	Promotes angiogenesis and neurogenesis in hippocampal neurons through modulation of pathways related to VEGF expression and PI3K/Akt signaling, both relevant to neuronal survival. In rodent models, this peptide reduces oxidative stress, neuroinflammation, and amyloid-β accumulation, suggesting a protective role against neurodegeneration [425,426].
*B. martensii* Karsch	BmK CT	A small peptide (~36–40 amino acids) structurally related to chlorotoxin (chlorotoxin-like peptide) and capable of crossing the Blood–Brain Barrier (BBB).	Inhibits matrix metalloproteinase 2 (MMP-2), an enzyme overexpressed in neuroinflammation and tumors. Exhibits anti-inflammatory properties in models of neuroinflammation by suppressing IL-6 and TNF-α [427]. As chronic neuroinflammation is driven by cytokines and MMPs contributes to neurodegeneration, BmK CT could mitigate these pathways.
B. martensii *Karsch*	BmK NSPK (Neuroprotective scorpion peptide Karsch	A low-molecular-weight peptide the structure of which has yet to be solved.	Shows CNS bioactivity without the neurotoxicity of classic neurotoxins. As part of its protective mechanism of action, BmK NSPK improves mitochondrial membrane potential and ATP production in primary cortical neurons, and inhibits Aβ-induced neuronal apoptosis by regulating Bcl-2/Bax ratios and caspase-3 activity [428].
B. martensii *Karsch*	BmK AGAP (Analgesic-antitumor peptide)	A 66-amino acid peptide originally isolated for its analgesic and anti-tumor effects.	Suppresses microglial activation and reduces secretion of pro-inflammatory cytokines (IL-1β, TNF-α, IL-6). Inhibits p38 MAPK and ERK^1/2^ signaling pathways, key regulators of inflammatory and apoptotic signaling in activated microglia [413].

## Data Availability

No new data were created or analyzed in this study. Data sharing is not applicable to this article.
